# The Effects of Cooperative Compliance on Firms’ Tax Risk, Tax Risk Management and Compliance Costs

**DOI:** 10.1007/s41471-021-00108-6

**Published:** 2021-03-23

**Authors:** Eva Eberhartinger, Maximilian Zieser

**Affiliations:** Vienna University of Economics and Business, Welthandelsplatz 1, 1020 Vienna, Austria

**Keywords:** Tax risk, Tax certainty, Risk management, Cooperative compliance, Compliance costs, Horizontal monitoring, G32, H21, H25, H83

## Abstract

In cooperative compliance programs, firms and tax administrations agree on cooperation instead of confrontation. Firms provide full transparency and advanced tax control frameworks. Tax administrations, in turn, offer certainty as to the tax treatment of complex transactions. In this study, we test how firms’ perceptions of tax risk, the quality of tax risk management, and compliance costs are related to cooperative compliance. To our knowledge, this is the first study that attempts to analyze both reasons for and consequences of participation in cooperative compliance programs. We examine the Austrian cooperative compliance pilot project known as horizontal monitoring that was aimed at large businesses and launched in 2011. We use survey data from representatives of firms participating in the pilot project and a sample of comparable firms under a traditional ex-post audit regime. We conduct group comparisons to test differences between these groups, as well as mediation analyses to shed light on more complex relationships between variables. Results show that horizontal monitoring firms perceive a significantly higher increase in tax certainty, which is associated with significant relative decreases in tax risk and compliance costs. Furthermore, while the quality of tax risk management upon entering the pilot project appears significantly higher for horizontal monitoring firms, they do not report greater improvement in tax risk management compared to the control group. These results are relevant for the development of cooperative compliance programs and the decision to participate in them.

## Introduction

We test whether cooperative compliance (CC), designed as a trust-based alternative to conventional tax audits, is an effective tool to decrease firms’ tax risk, to foster the quality of their tax risk management (TRM), and, at the same time, to reduce firms’ compliance costs. To this end, we consider the Austrian setting, where the CC pilot project known as horizontal monitoring (HM) was carried out from 2011 to 2018 and subsequently integrated into Austrian law in 2019. We compare survey responses from firms taking part in the pilot project with responses from comparable firms not participating in HM. Our results suggest that HM has decreased perceived tax risk (referring to the likelihood and magnitude of unexpected, adverse tax outcomes) and compliance costs by strongly increasing perceived tax certainty (referring to the certainty that tax authorities will not challenge current tax positions). Furthermore, we find evidence that firms reporting more developed tax-risk management systems were more likely to select and be accepted into the HM pilot project.

According to the OECD (2008), cooperative alternatives to tax audits have been developed primarily as a response to growing concerns of governments and the public about tax avoidance by large businesses. Based on the concept of “enhanced relationships” (OECD 2008), the OECD Forum on Tax Administration published an updated framework known as cooperative compliance (OECD 2013). It describes how tax administrations and taxpayers can have ongoing, trust-based relationships instead of the traditional, confrontational approach of ex-post tax audits. CC requires firms to employ advanced tax control frameworks and to be fully transparent regarding transactions, financial records, and other tax-related issues. In turn, tax administrations are expected to behave predictably and provide timely legal certainty to participating firms. Indeed, surveys conducted with Austrian firms confirm the central role of tax certainty, i.e., early agreement between tax authority and firm on the appropriate tax treatment of specific cases and circumstances, to avoid later disagreement and litigation (Enachescu et al. 2019).

Viewing CC from a principal-agent perspective, with the tax administration assuming the principal’s role and a firm’s management that of the agent, we propose that the reduction of agency conflicts is the primary goal of CC. Under conventional audit regimes, tax administrations exert control over firms’ management by enforcing tax law with the help of tax audits and litigation, often with long delays between actual transactions, the detection of questionable tax positions, and the resolution of legal conflicts. In a CC program, by contrast, both parties are expected to behave transparently and reduce information asymmetries between management and tax administration, which may stem from both management’s tax decisions or administration’s interpretation and application of tax law. Aligning the firm’s tax risk behavior with the tax administration’s preferences, CC may thus directly affect firms’ governance structure. From this, tax administrations expect increased and timely tax compliance. Ultimately, a tax administration may be able to re-allocate its resources and focus on the audit of high-risk taxpayers.

A reduction of information asymmetries and agency costs may reduce costs incurred not only by the tax administration but also by firms, which are expected to benefit from an improved relationship with tax authorities, resulting in immediate certainty as to the correct treatment of difficult tax questions. This stands in stark contrast to the conventional ex-post audit context, in which clarification of questions may take several years and cause a financial burden for firms. Furthermore, participation in HM should be more likely for firms with already advanced tax risk management systems and lower tax risk because such firms are most likely to benefit from a reduction of agency costs relative to costs associated with CC participation.

Against this background, this paper aims to assess whether CC fulfills its expectations. Specifically, we investigate if CC is associated with lower tax risk, better tax risk management, and lower compliance costs. We understand tax risk as the likelihood and magnitude of unexpected tax outcomes that can adversely affect the firm. We follow Brühne and Schanz (2019) in their definition of tax risk management as the entirety of a firm’s actions, tools, and processes implemented to prevent, mitigate, and control corporate tax risk exposure. Compliance costs comprise all costs incurred by the firm to comply with legal and administrative requirements in a tax context, including the process of determining taxes payable.

We expect the relationship between CC, tax risk, and tax risk management to be triangular. While we expect CC to influence tax risk and tax risk management, tax risk, and the state of tax risk management may also be regarded as important determinants of self-selecting or being admitted to CC. We thus expect that firms with low tax risk exposure and advanced tax risk management are more likely to participate in CC than high tax-risk firms with less developed tax risk management. At the same time, we also expect that CC participation further reduces tax risk and improves tax risk management.

To test these expected relationships, we compare survey responses from firms taking part in the pilot project (i.e., the treatment group) with responses from comparable firms not participating in the pilot project (i.e., the control group). We choose the Austrian implementation of CC, known as horizontal monitoring (HM), which closely follows the OECD recommendations for CC programs. From 2011 to 2018, HM was implemented as a pilot project, in which 13 large Austrian firms participated at the time of our study.

We conduct a survey in which 9 of the 13 firms participating in HM completed the questionnaire. As a control group, we invited 92 large non-HM firms that engage in tax policy, of which 31 completed the questionnaire. Although the number of respondents—especially in the subsample of HM firms—is small, we cover the majority of HM firms in Austria, and all participants are senior, experienced tax managers. We thus expect that our results are representative of the population of Austrian HM firms and that they may be informative for other countries as well.

Using questionnaire items based on current literature, we inquire about the perceived magnitude of current overall tax risk and tax risk management quality on a 7-point Likert-type scale. We also inquire about the perceived direction and magnitude of changes since entering HM (or during the past years for the control group), again on a 7-point scale, namely regarding the perceived change in tax risk, tax risk management quality, tax certainty, and compliance costs.

Our empirical hypotheses and analyses follow the expectations described above. To test group differences, we conduct group comparisons using non-parametric and parametric tests. We further rely on mediation analyses to test more complex relationships between variables. Although we cannot directly assess differences in tax risk and tax risk management that may have led to HM participation, we examine how much of the differences in current tax risk management and tax risk are explained (mediated) by perceived changes and to what extent these differences might be explained otherwise, in particular by pre-HM differences in these variables. In this way, we hope to shed more light on the potential selection or self-selection of firms into HM.

We find that the perceived increase in tax risk is significantly smaller for HM firms, which can be explained, in part, by a drastic perceived increase in tax certainty compared to the control group, confirming the importance of certainty found in a previous study with Austrian HM firms (Enachescu et al. 2019). We also find that HM firms report significantly lower current tax risk. This difference, however, cannot be explained by a mediation via the perceived decrease in tax risk, suggesting alternative explanations for this difference. Regarding tax risk management, HM firms also report significantly better current tax risk management quality, but no additional improvements due to HM participation. Better tax risk management may thus have been a decisive factor for the likelihood of HM participation. Regarding compliance costs, we find that HM firms report a significantly lower increase in costs—an effect that appears to be mediated by the reduction in tax risk and the increase in tax certainty.

Despite the small sample size, we find significant and strong differences and associations, in particular concerning the perceived change in certainty and tax risk. We confirm results using non-parametric methods such as U‑tests and Pearson correlations, as well as robustness tests in which we compute models removing the most influential observation from the sample.

Exploring additional responses collected in the survey, we find that the perceived changes in tax risk are also reflected in more specific types of tax risk, such as litigation risk or reputational risk. With regard to current sources of tax risk, we find that HM firms indicate less compliance risk and marginally higher operational risk. While HM firms do not report a significantly larger increase in tax compliance than the control group, they perceive a much greater improvement in their relationship with tax authorities. Regarding tax risk management methods, we find that HM firms rely more on advance informal agreements. Together with the improved relationship with the Austrian tax administration, this may be one important way by which HM increased tax certainty.

With CC becoming increasingly popular, systematic assessments of such programs are vital. However, the effects of CC programs on the firm have been the subject of little analysis. Despite the central role that tax risk, tax risk management, and compliance costs play in CC programs, there is very little empirical evidence regarding how they are related to CC. To the best of our knowledge, we are the first to bridge this gap and investigate the reasons for and the consequences of CC participation on firms’ tax risk, tax risk management, and compliance costs by directly comparing CC firms with firms under a conventional audit regime. By providing important evidence on the effectiveness of CC in reducing information asymmetries and agency costs, we hope to facilitate the decision to participate in HM and to contribute to the further development of CC initiatives.

## Related Background and Literature

### Cooperative Compliance

Cooperative compliance (CC) describes a family of alternative approaches to tax auditing that focus on cooperation and transparency. In such programs, firms generally commit to being completely transparent and improving their tax risk management. In exchange, tax administrations usually provide increased certainty and accelerated feedback about complex tax issues. CC can thus be described as “transparency in exchange for certainty” (OECD 2013, p. 28).

CC programs typically aim at saving resources, namely reducing the workload on the side of revenue bodies and reducing compliance costs for firms, while at the same time reducing potentially aggressive tax planning and ensuring compliance with tax laws. CC has proved to be popular around the world. In its 2013 report, the OECD mentions 24 countries that had implemented CC at that time. In most countries, a well-established tax control framework is now a requirement for joining a CC program (OECD 2016).

The development of the concept appears to follow in the wake of more service-oriented concepts of public administration, such as “new public management” or “new governance” (Ford and Condon 2011; de Widt 2017). Other theoretical influences of cooperative compliance lie in the so-called slippery-slope framework (Kirchler et al. 2008) and in “responsive regulation” (Braithwaite 2002). According to the slippery-slope framework, tax administrations’ power (i.e., audits and fines) and trust in tax administrations increase compliance, suggesting a balanced mix of coercive and cooperative trust-building measures. Similarly, responsive regulation describes how tax administrations should react to a heterogeneous population of taxpayers. It suggests that harsh audits and fines are appropriate only for taxpayers that are intrinsically reluctant to follow the law. For the majority of taxpayers, however, compliance can be improved with measures that foster self-regulation, such as services and education (Braithwaite 2002).

Overall, cooperative compliance programs aim to create a win-win situation for firms and tax administrations. De Simone et al. (2013) support this notion analytically. They model the relationship between tax authorities and taxpayers and find that enhanced relationship programs are mutually beneficial under certain conditions, including that reviewing firms’ tax positions in CC is not more expensive than in an ex-post audit scheme and that the overall cost of the program is low.

#### Austrian Horizontal Monitoring Project

Inspired by the OECD and by the Dutch cooperative compliance project,^1^ Austria introduced the HM pilot project in 2011 as part of the Fair Play Initiative of the Austrian Ministry of Finance (Schrittwieser and Woischitzschläger 2014; Elmecker et al. 2016). The Austrian HM project closely follows the recommendations laid out in the OECD CC framework (2008, 2013) and emphasizes the goal to create a win-win situation for firms and the Austrian tax administration (Elmecker et al. 2016).

The Austrian HM pilot project had several explicitly stated objectives: It was aimed at (i) fostering tax compliance, (ii) ensuring legally valid and timely tax collection, and, as a medium-term objective, (iii) shifting the resources of tax authorities towards high-risk taxpayers. As advertised benefits for companies, it aimed to (iv) reduce compliance costs and (v) promote legal certainty and planning security (Elmecker et al. 2016).

The pilot project was directed exclusively at large firms^2^ (i.e., turnover of more than 40 million Euro) falling under the responsibility of the Large-Business Unit (Großbetriebsprüfung) of the Austrian Ministry of Finance, who had their financial statements audited and certified. As a general rule, large businesses are subject to continuous tax audits (i.e., each business year is audited with near certainty), albeit with years of delay between the initial tax assessments and tax audits.

Participation in HM was voluntary. For admission to the pilot project, firms had to demonstrate tax compliance in the past. They were also required to have either an existing tax control framework or to be willing to develop such a framework in cooperation with the tax administration. Acceptance into the pilot study by the Large-Business Unit was part of a negotiation process that considered not only the quality of the tax control framework and prior good governance, but also questions of feasibility for tax auditors, i.e., the complexity of the business, and the total amount of resources dedicated to the pilot. For instance, financial institutions or the largest Austrian production corporations/groups, which have highly complex structures and business models, were not admitted. In the end, 15 firm groups with about 150 individual firms as group-members were part of the pilot, 2 ended their participation early, and 13 remained in the project until the end of the pilot in 2016^3^ (Elmecker et al. 2016; Schrittwieser and Woischitzschläger 2014).

Participation in HM was only possible if all members of a firm group (as defined in Austrian tax law) participated in the program. Moreover, for each firm group, one employee was designated as the main HM contact person. We thus expect firms within each group to be strongly aligned regarding taxation and their experiences with HM. For the remainder of the paper, we refer to HM firm groups as “HM firms”.

The 13 participating firms cover a variety of sectors and business models. They include six from the production sector, four from trade, two from energy, and one from the services sector. Seven of the HM firms engage in business-to-business, and four in business-to-consumers. The two HM firms from the energy sector serve businesses and consumers. Seven of the HM firms are sub-groups with an international ultimate parent. For the remaining six, the ultimate parent is Austrian. Their long-term effective tax rates range from 14 to 33%, with an average of 23%.^4^ A time trend in the effective tax rates of HM firms is not observable, i.e., there is no indication that tax expense increased or decreased over the years that firms participated in HM.

Upon acceptance, companies underwent a final tax audit, after which both the taxpayer and the Austrian tax authorities signed a “declaration of intent”. In the subsequent regular HM process, managers and tax officers met regularly (usually quarterly or bi-annually) to discuss current tax issues (Schrittwieser and Woischitzschläger 2014; Stiastny 2015; Elmecker et al. 2016).

Strictly speaking, HM provided only a “soft” version of legal certainty. Any pre-clearance during the HM process was not legally binding. Theoretically, both parties could eventually challenge the outcome in court, and the HM process did not legally prevent later ex-post audits. However, the evaluation report suggests that both sides followed the spirit of the agreement and refrained from challenging the outcome of the HM process. The Austrian tax administration, therefore, regarded the HM pilot project as a success (Elmecker et al. 2016). In case a firm wanted legal certainty in a strict sense, legally binding advance rulings were still available and in use. Usage of binding advance rulings is similar among HM firms and non-HM firms, as indicated by our survey. See Sect. 5.4 for more detailed analyses on the importance of tax risk management methods.

Marking the conclusion of the pilot project, Austria fully implemented HM for large businesses in 2019.^5^ The new legal basis provides specific provisions regulating legal certainty and setting more explicit standards for tax control frameworks required for acceptance into HM.

#### Effects of Cooperative Compliance Programs

Several national CC projects have been analyzed by researchers and practitioners. Literature predominantly focuses on legal questions, national experiences with CC, and differences between CC programs (e.g., Dabner and Burton 2009; Påhlsson 2013; Bronżewska 2016; Colon 2017; Björklund Larsen et al. 2018; Brøgger and Aziz 2018; Potka-Soininen et al. 2018; Björklund Larsen and Oats 2019; Majdanska and Pemberton 2019; de Widt et al. 2019).

Only a small number of studies empirically investigate the effects of CC on firms. Huiskers-Stoop (2015) discusses the effectiveness of the Dutch HM project for medium-sized businesses in the Netherlands. She emphasizes that, due to potential self-selection, HM could merely be a “formalization” of already existing differences in tax attitude and behavior. Using a survey assessing firms’ general experiences and firms’ perceived effectiveness of HM, she finds that HM likely improved tax certainty and tax compliance and reduced compliance costs for firms. The Dutch Tax and Customs Administration (Belastingdienst 2017) presented a comprehensive evaluation of HM to the Dutch parliament. Using surveys, they find a positive association between HM and compliance.

An evaluation report of the Austrian HM pilot project, based on limited statistics, survey data, and workshops, indicates that the project largely succeeded in fulfilling its goals of providing certainty and increasing efficiency (Elmecker et al. 2016). Enachescu et al. (2019), using survey data from this evaluation, analyze HM firms’ and tax auditors’ perceptions of the Austrian HM project in context with organizational change processes. They find that legal and planning certainty is important to stakeholders and regarded as one of HM’s main advantages. However, they observe that CC appears to represent a challenging paradigm shift for the Austrian tax administration.

These studies focus mostly on compliance and certainty. While our study also investigates the importance of tax certainty in the context of CC, it focuses on firms’ tax risk and tax risk management to assess how tax administrations affect firms’ governance. Further, we use mediation analysis to disentangle the reasons for and the consequences of HM participation in more detail.

Other studies also highlight the importance of increased tax certainty and predictability for CC firms (Boll and Brehm Johansen 2018; Goslinga et al. 2019). In an empirical analysis using confidential data from the IRS, Beck and Lisowsky (2014) analyze the effect of the US Compliance Assurance Process (CAP) on FIN48 tax reserves, a proxy for tax uncertainty disclosed in financial statements. They find that CAP participation is especially likely for firms with medium pre-CAP tax uncertainty. They also show that firms that participate in the scheme indeed reduce their FIN48 tax reserves.

Overall, little is known about the relationship between potential causes and effects of HM participation. To our knowledge, there is no empirical study attempting to disentangle the two central requirements and motivations for CC participation, namely tax risk, and tax risk management, and the effects CC has on these variables.

### Cooperative Compliance from a Principal-Agent Perspective

Cooperative compliance programs represent a significant change in the relationship between tax administrations and taxpayers. Its apparent popularity can be explained by an underlying desire to reduce information asymmetries between firms and tax administrations. Viewing CC from the perspective of a principal-agent setting with self-protection (see Biswas et al. 2013) could explain the success of CC and strengthens theoretical predictions about its effects on tax risk, tax risk management, and compliance costs.

In a classic principal-agent setting (Jensen and Meckling 1976), tax administrations have no role. With regard to taxation, Schön (2008, p. 34), for instance, asserts that while managers are obliged to administer the tax affairs of firms, they do so as part of the duties they owe to the shareholders, not to tax administrations which represent the state and its government. In tax matters, management thus serves the interests of the shareholders. In this regard, a stream of literature discusses the conflicting interests of the shareholder and the management with regard to tax avoidance or tax evasion (Crocker and Slemrod 2005; Chen and Chu 2005; Desai and Dharmapala 2006; Phillips 2003). Phillips and Sansing (1998) rely on the principal-agent framework to describe the contract between the taxpayer and tax practitioner.

In contrast to this classic interpretation of the shareholder as a principal and management as the agent, the theory may also explain the conception of cooperative compliance. Some scholars (mostly in the context of the determination of commercial and taxable profit) hold that the state’s stake in a firm is similar to that of the shareholder (Döllerer 1988; Moxter 1997; Euler 1998). The state participates in a firm’s profits and losses and is interested in not overly exploiting its funds. In such a setting, the state takes the principal’s role, similar to a shareholder, which is entitled to a share in the firm’s profit (i.e., in the form of taxes; Reinganum and Wilde 1985).

In this principal-agent setting, information asymmetries may arise from the behavior of either party. For instance, information asymmetries may relate to management’s choices and decisions that affect the tax liability, particularly tax planning activities. Information asymmetries can also stem from tax administrations’ interpretation and application of tax law in specific cases. The state, therefore, uses tools to reduce information asymmetries and conflicts with firm management. In a conventional confrontational setting, these tools usually comprise enforcement by regular tax audits and legal proceedings, causing high “agency costs” on both sides.

By contrast, CC requires firms to employ and improve internal tax risk management and to disclose their tax strategy and transactions in real time. Transparent behavior by both the firm and the tax administration in the form of early tax certainty, i.e., the reduction of information asymmetries for both sides simultaneously, may thus align the agents’ tax risk behavior with the principal’s preferences and ensures that a firm’s management does not unduly reduce the state’s share of the profit.

Therefore, the way tax administrations act towards firms affects firms’ governance structure (see Desai and Dharmapala 2008; Schön 2008). Furthermore, in line with signaling theory (Spence 1973, 2002), firms that engage in CC can more easily signal to tax authorities an attitude which is more in line with the public interest of following the “spirit of the law” (OECD 2013). However, to be an attractive (voluntary) alternative to conventional tax audit regimes, CC should ultimately lead to a reduction in agency and signaling costs for both firms and tax administrations.

### Tax Risk and Tax Risk Management

We regard tax risk as the likelihood and magnitude of unexpected tax outcomes that can adversely affect the firm. Literature shows similar definitions: Neuman et al. (2020), for example, define tax risk as “*the uncertainty about future tax outcomes*”, which can stem from (i) economic risk, (ii) tax law uncertainty, and (iii) inaccurate information processing. Similarly, Neubig and Sangha (2004) define tax risk as “*the likelihood and magnitude of outcomes that are different than expected*” (p. 114). Emphasizing its potentially negative effects, Ernst & Young describe tax risk as something that “*either adversely affects the company’s tax or business objectives or results in an unanticipated or unacceptable level of monetary, financial statement or reputation exposure*” (Ernst & Young 2006; quoted in Mulligan and Oats 2009, p. 685).^6^ More recently, Brühne and Schanz (2019) find in an interview study that definitions of tax risk differ among practitioner groups, with firm insiders perceiving solely the downside potential. They identify six tax risk components: Financial risk, compliance risk, reputational risk, tax process risk, political risk, and personal liability risk.

In line with Brühne and Schanz (2019), we define tax risk management “*as the entirety of a firm’s actions, tools, and processes implemented to prevent, mitigate, and control corporate tax risk exposure*”. The inclusion of tax risks in risk management systems results from increasing public awareness and regulatory attention. A worldwide survey by Ernst & Young (2004), for example, identified a change in the role of tax directors. The reasons for this change lie in “*increased scrutiny of companies’ tax issues by regulators, legislators, tax authorities, and the media; increased interest in corporate tax policy by shareholders, audit committees, and management; and an overall focus on transparency and disclosure, which itself is a direct result of such mandates as the Sarbanes-Oxley Act in the United States* […]* and the European Union’s* […]* 8th Directive*” (p. 1). Since then, the focus on tax has increased further, as demonstrated by the public debate about taxation and domestic as well as international efforts to combat tax avoidance and profit shifting.

Increased focus on risky tax positions resulted in demand for tax risk management, which was met by consulting firms. PricewaterhouseCoopers, for example, developed a tax risk management model based on COSO’s internal control framework (Elgood et al. 2004). Nonetheless, the degree of implementation by firms of tax risk management still varies. Brühne and Schanz (2019) find in their interview study that, in fact, tax risk management is not well integrated with the general risk management system of the firm. Lavermicocca and McKerchar (2013) conclude that only few Australian firms have a tax risk management system in place. They find that for firms that employ tax risk management systems, the level of acceptable tax risk is reduced, and the level of income tax compliance improves. Wunder (2009) analyses the state of tax risk management in the United States and abroad. She identifies transactional risk from M&A activities as the most prominent type of tax risk for firms. Other research on tax risk management mostly focuses on managerial aspects (e.g., Plesner Rossing 2013; Plesner Rossing and Rohde 2014).

One tool to mitigate tax risk is the establishment of a tax control framework, which is a central requirement for firms to participate in CC programs. Tax control frameworks are thus a substantial part of the tax risk management system, which is one aspect of the overall risk management system and the governance structure of firms (Whait 2012; OECD 2013, Chap. 4; Colon and Swagerman 2015; van der Enden and de Groot 2015; van der Hel-van Dijk and Siglé 2015). Seen from the perspective of tax administrations, a well-organized tax control framework reduces the risk of fraud, tax evasion, and possibly even tax avoidance (Freedman et al. 2009; Mulligan and Oats 2009). Siglé (2019), using survey data and tax compliance statistics, conducted a detailed analysis of HM-related variables and their effects on tax compliance. While the quality of the tax control framework is positively related to taxpayer transparency and the quality of the working relationship with the tax administration, the author only finds little evidence on the effects of tax control frameworks and transparency on tax compliance. Goslinga et al. (2019), however, find that for firms, the quality of tax control frameworks is positively associated with the need for certainty and the perceived importance of tax compliance.

## Hypotheses Development

Based on the characteristics of HM and on the principal-agent setting, we assume that the primary goal of HM is the reduction of information asymmetries and agency conflicts by requiring firms to utilize a tax control framework and by increasing transparency. For firms, this should lead to a reduction of uncertain tax outcomes, which may ultimately lead to a reduction in compliance costs.

Besides differences in these variables between HM firms and non-HM firms, we thus expect indirect (i.e., mediation) effects of HM on tax risk and on compliance costs via certainty. Moreover, because tax risk and tax risk management may represent both cause and effect of HM participation (i.e., firms may self-select into HM), we also formulate mediation hypotheses to disentangle these relationships.

HM may achieve a reduction in tax risk both by requiring firms to behave more transparently, as well as by providing early certainty for participating firms. Because of timely clarification of tax issues in HM, we expect HM firms to perceive improved tax certainty. As tax certainty, i.e., certainty about the sustainability of tax positions, is discussed as an important determinant of tax risk, we expect the improvement of tax certainty to be a significant driver of tax risk reductions. A remaining direct effect of HM on tax risk unexplained by changes in certainty may be attributed to other, unobserved reasons (e.g., higher transparency). We hypothesize:

### H1

Compared to non-HM firms, HM firms report a larger reduction (or a smaller increase) in perceived tax risk.

### H1a

The negative association between HM and changes in perceived tax risk is mediated by increased perceived tax certainty (relative to non-HM firms).

Agency costs (i.e., compliance with enforcement measures) should be comparatively more costly to firms with inherently little tax risk. Due to much stricter standards regarding the transparency of firms, we thus expect that firms with lower tax risk are more likely to enter HM. In addition, improvements in tax risk should be reflected in the current tax risk. For both reasons, we expect HM firms to report a smaller current tax risk than non-HM firms. Both tax risk before HM participation, as well as the hypothesized reductions of tax risk during HM, may explain lower current tax risk reported by HM firms. To assess whether lower current tax risk in HM firms is associated with HM only via improvements in tax risk or via other, unobserved differences (e.g., pre-HM tax risk), we expand H2 by this mediation effect:

### H2

Compared to non-HM firms, HM firms report a smaller perceived current tax risk.

### H2a

The association between HM and lower perceived current tax risk is mediated by reductions in perceived tax risk (relative to non-HM firms).

A tax control framework can be seen as an important measure to align the interests of firms with those of tax administrations. As a requirement for entering HM, firms either had to have a tax control framework in place or had to be prepared to develop it in cooperation with the tax administration. For both reasons, we expect tax risk management to be more elaborated in HM firms than in the control group. Because tax administrations may also support subsequent improvements of the tax control framework in HM firms, we expect HM to lead to higher tax risk management quality, as perceived by participants, during HM participation. Both tax risk management quality before HM participation and the hypothesized improvements of tax risk management during HM may explain better current tax risk management quality reported by HM firms. To assess whether higher current tax risk management quality in HM firms is associated with HM only via improvements in tax risk management, or via other, unobserved differences (in particular, pre-HM tax risk management), we expand H3 by this mediation effect:

### H3

Compared to the non-HM firms, HM firms report a higher perceived current TRM quality.

### H4

Compared to non-HM firms, HM firms report a larger improvement in perceived TRM quality.

### H3a

The association between HM and higher perceived current TRM quality is mediated by perceived improvements in TRM (relative to non-HM firms).

The effect of HM on compliance costs may be twofold. We assume that HM reduces overall compliance costs for firms because early clarification of tax matters may allow firms to avoid later tax audits and tax disputes and may reduce the need for tax consulting services, thereby reducing agency costs on the side of HM firms. On the other hand, HM requires advanced tax risk management that can increase compliance costs. In aggregate, we expect that compliance costs are lower in HM because we assume that voluntary participation in such a program is motivated, at least in part, by an overall cost reduction. A reported cost reduction through HM may be mediated by the perceived increase in tax certainty and the perceived decrease in tax risk. A (marginal) increase in compliance costs, however, may be mediated by better tax risk management. Therefore, we expand H5 by these mediation effects:

### H5

Compared to non-HM firms, HM firms report a larger perceived reduction (or smaller increase) in compliance costs.

### H5a

The association between HM and lower perceived compliance costs is mediated by the increase in perceived tax certainty (relative to non-HM firms).

### H5b

The association between HM and lower perceived compliance costs is mediated by the reduction of perceived tax risk (relative to non-HM firms).

### H5c

The association between HM and (marginally) higher perceived compliance costs is mediated by an improvement in perceived TRM quality (relative to non-HM firms).

## Method

### Data Collection and Participants

All 13 HM firms consented to public identification (Elmecker et al. 2016, p. 21).^7^ The survey was therefore addressed to all 13 firms participating in HM at the time of the survey, namely to the individuals who manage their firms’ HM process. They serve as the main contact to tax administration, and we expect them to have the best knowledge about the program within each firm.

To address our control group, namely firms that were subject to traditional ex-post tax audits, we invited heads of tax of 92 firms that were members of the Tax Policy Group in the Federation of Austrian Industries.^8^ We expect all participants to be experienced in tax matters and in senior positions.^9^ The Austrian Ministry of Finance and the Federation of Austrian Industries provided support with identifying potential participants and sending out invitations. Data collection took place between October 2017 and March 2018. We used follow-up phone calls and e‑mails as reminders to increase participation.

We acknowledge that the selection of our participants into HM firms is likely endogenous: HM firms self-select into participation in the pilot and are accepted into the program by the Austrian tax administration. According to the criteria for participation in the pilot, HM firms must have demonstrated prior tax compliance and (the willingness to develop) advanced TRM systems. Non-HM firms, however, may or may not have been compliant in the past and may differ with regard to tax risk management. With regard to our control group, firms self-select into membership of the Federation’s Tax Policy Group, which includes predominantly production firms, but also financial industry, infrastructure, and related services. By asking about both present tax risk and TRM quality as well as perceived changes therein, we attempt to shed more light on the reasons for and consequences of HM participation.

Table 1 shows the number of invited firms and response rates. Some participants did not complete the questionnaire. In our main analyses, we only use data from participants who answered all items of interest. We achieved high response rates in both groups, with 9 of 13 invited HM firms and 31 of 92 invited non-HM firms completing the full questionnaire.

**Table 1:** Number of invited firms and responses by group

	Firms in horizontal monitoring (HM firms)	Firms in ex-post tax audit (non-HM firms, control group)
Total number of HM firms	13	–
Firms invited	13	92
Completed questionnaires	9 (69%)	31 (34%)
Support	Austrian Ministry of Finance	Federation of Austrian Industries

#### Responding Firm and Participant Characteristics

Overall, we find that responses by the control group and HM firm do not differ significantly in variables measuring general firm or individual characteristics. *χ*^*2*^-tests and U‑tests show no significant differences between the two groups’ distributions of answers concerning gender, age, and position within the firms (see Table 2 for response frequencies and results of the statistical tests). We find that participants in both groups are predominantly male. The sample mostly consists of experienced, senior experts: 88% of participants are older than 35; 85% are tax director or a position senior thereto, including 28% at board level. This high expertise indicated by participants substantiates our expectation that participants are experts who can give reliable assessments of their firms’ tax risk and governance.

**Table 2:** Sociodemographic data by group

	HM firms (*n* = 9)		Control group (*n* = 31)		Total sample (*n* = 40)		Comparisons	
	*n*	%	*n*	%	*n*	%	*p (χ*^*2*^*)*	*p (U)*
*Gender*							0.495	–
Female	1	11.1	3	9.7	4	10.0		
Male	6	66.7	26	83.9	32	80.0		
No answer	2	22.2	2	6.5	4	10.0		
*Position*							0.195	–
Chief executive officer	0	0.0	3	9.7	3	7.5		
Chief financial officer	1	11.1	7	22.6	8	20.0		
Head of accounting	4	44.4	5	16.1	9	22.5		
Tax director	3	33.3	11	35.5	14	35.0		
Tax manager	1	11.1	0	0.0	1	2.5		
Other	0	0.0	2	6.5	2	5.0		
No answer	0	0.0	3	9.7	3	7.5		
*Age*							0.748	0.936
25–34	0	0.0	1	3.2	1	2.5		
35–44	3	33.3	11	35.5	14	35.0		
45–54	2	22.2	11	35.5	13	32.5		
55–64	2	22.2	5	16.1	7	17.5		
>64	0	0.0	1	3.2	1	2.5		
No answer	2	22.2	2	6.5	4	10.0		

This table shows participants’ responses to sociodemographic questions by group and results from *χ*^*2*^*-* and U‑tests. “No answer” is included in *χ*^*2*^*-tests* because it was provided as an answer option and chosen by a non-trivial number of participants. The comparison column shows *p*-values of exact *χ*^*2*^*-*tests, which test whether the distribution of answer frequencies is independent of the group, and of U‑tests when applicable.

Participants also provided details about their firms’ size and organizational setting (see Table 3 for response frequencies and *χ*^*2*^- and U‑tests). Statistical tests do not indicate significant differences between the answers regarding firm characteristics, i.e., the number of tax jurisdictions the firm is subjected to, worldwide sales, whether the firm is publicly listed on the stock market, whether the firm is part of a group (a group operating only within Austria or an international group) and the residence country of the group parent.

**Table 3:** Firm characteristics by group

	HM firms (*n* = 9)		Control group (*n* = 31)		Total sample (*n* = 40)		Comparisons	
	*n*	%	*n*	%	*n*	%	*p (χ*^*2*^*)*	*p (U)*
*No. of tax jurisdictions*							0.435	0.124
2–5	4	44.4	7	22.6	11	27.5		
6–10	3	33.3	11	35.5	14	35.0		
11–20	0	0.0	2	6.5	2	5.0		
>21	1	11.1	10	32.3	11	27.5		
No answer	1	11.1	1	3.2	2	5.0		
*Firm sales in Austria (million euro)*							0.788	0.746
0.7–10	0	0.0	2	6.5	2	5.0		
10–40	0	0.0	1	3.2	1	2.5		
40–250	3	33.3	6	19.4	9	22.5		
250–1000	1	11.1	9	29.0	10	25.0		
>1000	4	44.4	11	35.5	15	37.5		
No answer	1	11.1	2	6.5	3	7.5		
*Publicly listed on the stock market*							0.669	–
Yes	4	44.4	18	58.1	22	55.0		
No	3	33.3	9	29.0	12	33.0		
No answer	2	22.2	4	12.9	6	15.0		
*Company part of a group*							0.397	–
Yes, an Austrian group only	0	0.0	1	3.2	1	2.5		
Yes, an international group	7	77.8	28	90.3	35	87.5		
No	2	22.2	2	6.5	4	10.0		
*Worldwide group sales (million euro)*							0.355	0.593
0.7–10	0	0.0	1	3.2	1	2.5		
40–250	0	0.0	1	3.2	1	2.5		
250–1000	0	0.0	2	6.5	2	5.0		
>1000	5	55.6	23	74.2	28	70.0		
No answer	4	44.4	4	13.0	8	20.0		
*Residence of group parent*							0.863	–
Austria	3	33.3	14	45.2	17	42.5		
Germany	2	22.2	5	16.1	7	17.5		
Switzerland	0	0.0	1	3.2	1	2.5		
United Kingdom	1	11.1	1	3.2	2	5.0		
United States	0	0.0	2	6.5	2	5.0		
No answer	3	33.3	8	25.8	11	27.5		

This table shows participants’ responses to questions regarding their firms’ characteristics and results from *χ*^*2*^*-* and U‑tests. “No answer” is included in *χ*^*2*^*-tests* because it was provided as an answer option and chosen by a non-trivial number of participants. The comparison column shows *p*-values of exact *χ*^*2*^*-*tests, which test whether the distribution of answer frequencies is independent of the group, and of U‑tests when applicable.

To guarantee anonymity, in particular for the small group of HM firms, we did not inquire about additional details. While we know which firms participated in HM, we cannot identify the firms who answered our questionnaire. It is reasonable to assume that in both groups, the majority of firms is from the production industry because the majority of the 13 HM firms invited to reply, as well as the majority of members to the Federation of Austrian Industries, where we recruited our control group, are in the production industry.

### Material

We conducted an electronic survey study among Austrian firms that, at the time, did or did not participate in the HM pilot project. Designing the survey, we incorporated input from semi-structured interviews with the head of Large-Businesses Unit in the Austrian Ministry of Finance, with the head of taxes of the Austrian subsidiary of a large multinational firm that participates in multiple cooperative compliance programs around the world, and with a senior academic specializing in the field.^10^ To avoid confidentiality concerns and reduce effort on the side of participants, we chose items that directly assess the subjective view of participants. Because the survey was aimed at tax experts, we are confident that all items were understood and interpreted correctly and were able to capture complex constructs such as “tax risk” and “tax certainty” reliably and efficiently. Pre-tests with 14 tax experts and scholars were able to confirm this.

Questionnaire items covered the tax risk of the firm and specifically asked about the perceived level of tax risk at the present time and the perceived change in tax risk. Additional items inquired as to perceived sources of tax risk, based on Mulligan and Oats (2009) and Wunder (2009), which include transactional risk, operational risk, compliance risk, financial accounting risk, management risk, reputational risk, and portfolio risk. In addition, items covered the tax risk management system of the firm, based on Lavermicocca and McKerchar (2013).

Most answers, apart from demographics, were given on a 7-point Likert-type scale, either expressing magnitude or agreement in general (1 to 7) or the magnitude and direction of change (−3 to +3). Demographic questions included group status, turnover, and residence country of firms, as well as gender, age, and position of respondents. To a limited extent, open questions were included. All questions and items used in the questionnaire are presented in Appendix B.

Because taxation is a sensitive area, we regarded anonymity as especially critical for the validity of our results. Participants were therefore guaranteed full anonymity, and responses were stored in an anonymized format with regard to both the firm and the individual. The number of demographic questions and their detail was limited to ensure that the identification of respondents or their firm is not possible. Furthermore, respondents could opt out of demographic questions.

To assess our hypotheses, we focus on items and scales which directly assess the constructs of interest (see Table 4), namely the currently perceived tax risk and tax risk management quality and the perceived changes therein, as well as perceived changes in tax certainty and compliance costs. Other scales and items are used in additional tests to shed more light on differences between HM firms and the control group.

**Table 4:** Survey items and scales used in the analyses

	Mean (*SD*)	Cronbach Alpha/Item-scale correlation
**Items and scales assessing tax risk and tax risk management at the time of participation**		
*CurrTaxRisk* (“How would you describe your company’s tax risk profile?”) (very low tax risk (1)–very high tax risk (7))	2.93 (1.23)	–
*CurrTRM* (3-item scale) (not at all (1)–to a great extent (7))	4.53 (1.52)	0.86
“Is the identification and management of tax risk in your company part of the overall risk management system?”	4.73 (1.84)	0.70
“Is your tax risk management system well documented?”	4.45 (1.68)	0.77
“Is your tax risk management system operationalized in daily business?”	4.40 (1.65)	0.74
**Items and scales assessing change**^**a**^ (strong decrease (−3)–no change (0)–strong increase (+3))		
*∆TaxRisk* (“Tax risk for your company”)	0.75 (1.58)	–
*∆Certainty* (“Tax certainty for your company”)	0.20 (1.90)	–
*∆Costs* (“Compliance costs of your company”)	1.05 (1.34)	–
*∆TRM* (3-item scale)	1.15 (0.81)	0.82
“Quality of the tax risk management system”	1.13 (0.85)	0.82
“Degree to which the tax risk management system is formalized (i.e., well documented)”	1.40 (1.06)	0.66
“Degree to which tax risk is included in the general risk management system”	0.93 (0.89)	0.59

^a^HM firms reported change since entering the HM program, the control group for the last 10 (*n* = 23) or 5 years (*n* = 8).

## Analysis and Results

### Variables

As variables for our main analysis, we use single-item values, as well as scale values calculated as the mean of multiple items. Table 4 presents scales and items used in the main analyses, including internal consistencies and item scale correlations for multi-item scales. All items were rated on 7‑point scales. The variables are as follows: *HM* indicates HM participation, with non-HM firms being assigned 0 and HM firms 1. For the current tax risk reported by participants (*CurrTaxRisk*), we asked how participants would describe their company’s tax risk profile on a scale from 1 (very low tax risk) to 7 (very high tax risk). For the perceived changes in tax risk (*∆TaxRisk*), tax certainty (*∆Certainty*), and compliance costs (*∆Costs*), respondents could indicate the perceived change from −3 (strong decrease) via 0 (no change) to +3 (strong increase). We focus on subjective perceptions for two reasons: first, hard facts are often not readily available or contain highly sensitive information; second, based on participants’ expertise, we can reasonably assume that responses are accurate and reliable.

With regard to tax risk management (TRM) quality, participants specified the perceived degree of integration of tax risk in the firm’s general risk management, the degree of documentation of the tax risk management system and the operationalization of tax risk management in daily business, all on a scale from 1 (not at all) to 7 (to a great extent), resulting in the 3‑item scale *CurrTRM*. For the three-item scale *∆TRM,* participants indicated the change in tax risk management quality, the change in the inclusion of tax risk in general risk management, and the change in the formalization of tax risk management, each on a scale from −3 (strong decrease) via 0 (no change) to +3 (strong increase). Both scales show good internal consistencies of α ≥ 0.82.

As the control variable *Years*, we use the time frame for which participants indicated change into the analyses of all dependent variables representing perceived change. Firms entered the HM program over the course of 4 years. We asked HM-firm participants to indicate changes since their firm entered the pilot project, which could potentially affect the magnitude of perceived changes. We thus asked participants to indicate in which year their firms entered the HM program in order to compute the years until survey participation (M = 5.7, SD = 0.82, Range = 4–7). For non-HM firms, to achieve variation in the number of years, we asked about perceived changes during either the past 5 years or the past 10 years.^11^ From this information, we calculated the variable *Years* for both groups.

### Descriptive Statistics and Group Differences

As the first step in our analysis, we compute group comparisons between HM firms and our control group as well as correlations between our variables of interest. Because of our small sample size, outliers, and potential violations of distributional assumptions associated with t‑Tests and Pearson correlations may affect estimates. Therefore, we also compute U‑tests and Spearman rank correlations, which are based on ranks generated from the original data and are robust to outliers and distributional violations. Table 5 displays descriptive statistics by group as well as group comparisons based on t‑Tests and U‑tests. Table 6 displays Pearson and Spearman correlation coefficients.

**Table 5:** Descriptive statistics and group comparisons

	HM firms (*n* = 9)				Control group (*n* = 31)				Comparisons	
Variables	Mean	*SD*	Median	Mean rank	Mean	*SD*	Median	Mean rank	Mean difference	Mean rank difference
*CurrTaxRisk*	2.00	0.87	2	11.28	3.19	1.12	3	23.18	−1.19***	−11.90***
*CurrTRM*	5.48	0.70	5.67	28.78	4.25	1.59	4.67	18.10	1.23**	10.68**
*∆TaxRisk*	−0.78	1.39	0	9.83	1.19^+++^	1.35	1	23.60	−1.97***	−13.76***
*∆Certainty*	2.22^+++^	0.83	2	33.00	−0.39	1.71	−1	16.87	2.61***	16.13***
*∆Costs*	0.22	0.67	0	13.33	1.29^+++^	1.40	2	22.58	1.07**	−9.25**
*∆TRM*	1.22^+++^	0.71	1	21.22	1.13^+++^	0.84	1	20.29	−0.09	0.93

This table displays means, medians, and mean ranks of our main variables of interest as well as group comparison of means and mean ranks. Significance levels are based on conventional independent t‑Tests (for mean differences) and on exact U‑tests (for mean rank differences). ***, ** and * indicate two-tailed statistical significance at the 1, 5, and 10% level, respectively. ^+^, ^++^, and ^+++^ denote means of items/scales measuring perceived change that are significantly different from zero at the 1, 5, and 10% level, respectively.

**Table 6:** Bivariate correlations

Variables	1.	2.	3.	4.	5.	6.	7.
1. *HM*	–	–	–	–	–	–	–
2. *CurrTaxRisk*	−0.41*** (−0.45***)	–	–	–	–	–	–
3. *CurrTRM*	0.34** (0.39**)	0.02 (−0.02)	–	–	–	–	–
4. *∆TaxRisk*	−0.53*** (−0.51***)	0.25 (0.33**)	−0.10 (−0.13)	–	–	–	–
5. *∆Certainty*	0.58*** (0.59***)	−0.29* (−0.32**)	0.40** (0.47***)	−0.56*** (−0.54***)	–	–	–
6. *∆Costs*	−0.34** (−0.36**)	0.24 (0.31*)	−0.15 (−0.22)	0.59*** (0.64***)	−0.46*** (−0.44***)	–	–
7. *∆TRM*	0.05 (0.03)	0.23 (0.22)	0.52*** (0.38**)	0.25 (0.28*)	0.01 (0.06)	0.14 (0.16)	–
8. *Years*	−0.51*** (−0.44***)	0.15 (0.16)	−0.39** (−0.43***)	0.45*** (0.42***)	−0.28* (−0.23)	0.30* (0.28*)	−0.12 (−0.09)

This table displays Pearson correlation coefficients and Spearman (rank) correlation coefficients (in parentheses) between variables used in the main analyses. *HM* is a dummy variable, with 1 assigned to HM firms and 0 assigned to the control group. ***, ** and * indicate two-tailed statistical significance at the 1, 5, and 10% level, respectively.

Both t‑ and U‑tests (see Table 5) yield very similar results and indicate that HM firms perceive significantly less current tax risk (*CurrTaxRisk*), lending support to Hypothesis H2. Tests also indicate that HM firms experienced significantly stronger reductions in tax risk (*∆TaxRisk*) and compliance costs (*∆Costs*), providing evidence for Hypothesis H1 and H5. Moreover, HM firms report significantly greater improvements in certainty (*∆Certainty*), which we hypothesize to be the main mechanism by which tax risk is reduced. This mediation effect formulated in Hypothesis H1a is tested in the following section. While HM firms perceive their current TRM systems to be of significantly higher quality (*CurrTRM*) than the control group, they do not appear to have experienced a larger improvement in *∆TRM*. We thus find evidence for Hypothesis H3, but not H4.

**Fig. 1:**
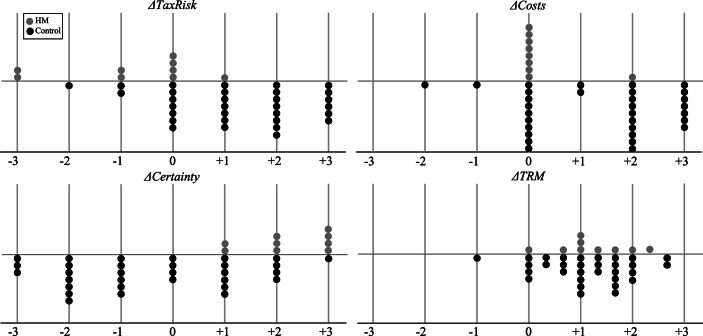
Responses to items measuring change. This figure presents responses by each participant from HM firms (*grey*) and the control group (*black*) in the four main variables measuring change from −3 (strong decrease) via 0 (no change) to +3 (strong increase). For variable definitions and item wordings, see Table 4.

With regard to perceived changes in tax risk and certainty, it is noteworthy that signs also point in opposite directions, with HM firms indicating an increase in certainty significantly different from zero, and the control group indicating an increase in tax risk significantly different from zero. To better illustrate the distribution in responses and differences between groups, Fig. 1 shows responses by each participant in variables measuring change.

Pearson and Spearmen rank correlations yield very similar results (Table 6), which suggests that estimates of differences between groups and associations between variables are largely unbiased from potential outliers or other distributional violations.

Correlations with the indicator variable *HM* (Table 6, column 1) reflect differences as indicated by t‑ and U‑tests. In terms of effect size, both Pearson and Spearman Correlations with *HM* indicate strong differences between HM firms and the control group, in particular with regard to *∆Certainty* and *∆TaxRisk*. Furthermore, we find a considerable negative correlation between *∆Certainty, ∆TaxRisk,* and *∆Costs.* A relatively weak correlation between *∆TaxRisk* and *CurrTaxRisk* suggests that the difference in current tax risk between HM firms and the control group cannot be explained solely by differences in *∆TaxRisk.*

While group differences and correlations provide insights into possible consequences of and reasons for HM participation, we conduct mediation analyses as outlined in the next section to shed more light on the interrelation between variables and to test the hypothesized mediation effects.

### Mediation Analyses

Mediation analysis of cross-sectional questionnaire data can give additional insights into associations between participants’ responses; we acknowledge that it allows no direct causal inferences. Despite our small sample size, we believe that these tests are valuable to better explain differences between HM firms and the control group and shed more light on firms’ potential self-selection into the HM program. We thus use the following analysis to further test our main Hypotheses H1 to H5 and potential mediation effects as formulated in Hypotheses H1a, H2a, H3a, and H5a–c.

For the mediation analyses, we use maximum likelihood estimation with robust standard errors using the sem function in STATA (version 16) in conjunction with the nlcom command to calculate indirect effects. We conduct the same analyses with the PROCESS macro for SPSS (Hayes 2020) using OLS estimation and bootstrapped standard errors for indirect effects (10,000 bootstrap samples), which results in virtually identical parameters and negligible differences in standard errors. To ensure that single outliers do not excessively drive results, we conduct robustness checks based on resampling, which are outlined at the end of this section.

**Fig. 2:**
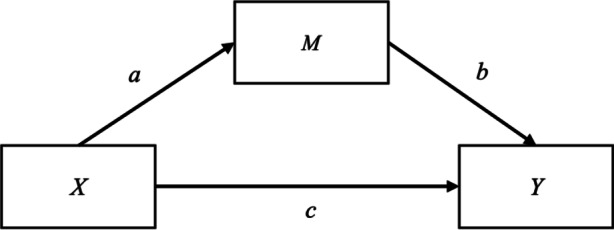
Illustration of the basic mediation model used in the analyses. *X*, *M* and *Y* represent the independent variable, the mediator variable, and the dependent variable, respectively. Arrows *a*, *b* and *c* represent the direct effects of *X* on *M*, of *M* on *Y* and of *X* on *Y* controlled for *M,* respectively. The indirect (mediated) effect of *X* on *Y* via *M* is measured by the product of *a* and *b*.

Mediation analysis is based on the decomposition of the total effect of an independent variable *X* on a dependent variable *Y* into a direct effect of *X* on *Y*, and an indirect effect of *X* on *Y* via *M* (see Fig. 2 for an illustration of the basic model). To this end, we estimate the effect of *X* on *M*, as well as the simultaneous effects of *M* and *X* on *Y*. The net effect *c* of *X* on *Y* (controlled for *M*) is the direct effect and is equivalent to the coefficient estimate in a multiple regression. The product a * b of the effect estimates of *X* on *M* and *M* on *Y* (with the latter controlled for *X*) equals the indirect effect of *X* mediated by *M*. The gross effect of *X* on *Y* (without controlling for *M*) is the total effect and corresponds to the sum of direct and indirect effects *c* *+* a * b in the basic model presented in Fig. 2.

In our analyses, we control all variables that measure perceived change for *Years*. For example, we analyze whether *∆Certainty* (M) mediates the effect of *HM* (X) on *∆TaxRisk* (Y), with both *∆Certainty* and* ∆TaxRisk* being controlled for* Years.* We thus test the mediation *HM* → *∆Certainty* → *∆TaxRisk.* As another example, we analyze whether *∆TRM* (controlled for *Years*) mediates the effect of *HM* on *CurrTRM.*

**Fig. 3:**
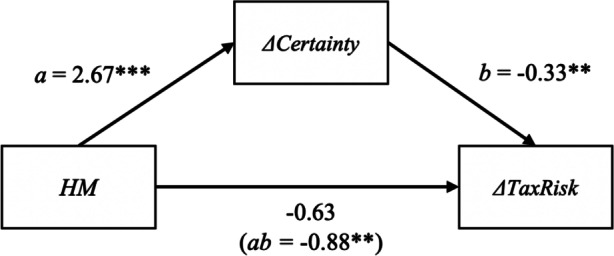
Illustration of the mediation model displayed in Table 7. Coefficients represent direct effects and the indirect mediation effect in parentheses. Effects on *∆Certainty* and* ∆TaxRisk* are controlled for *Years* (not depicted). ***, ** and * indicate two-tailed statistical significance at the 1, 5 and 10% level, respectively.

We find both a significant total effect of *HM* on *∆TaxRisk* and a mediated effect via *∆Certainty* (see Table 7 for model coefficients and Fig. 3 for an illustration of the mediation model). We thus find support for H1, because *HM* firms report a significantly smaller increase in perceived tax risk than the control group, expressed in a significantly negative total effect of −1.513 of HM of *∆TaxRisk*. Furthermore, these results support H1a, as a significant proportion of the difference in *∆TaxRisk* appears to be mediated by *∆Certainty,* rendering the remaining direct effect of *HM* on* ∆TaxRisk* non-significant. Much of this mediation effect can be attributed to a notable direct effect of *∆HM* on *∆Certainty* of 2.666, which is highly significant and, with responses being given on seven-point scales, qualitatively large. The control variable *Years* shows a significant (at the 10% level) association with *∆TaxRisk* when both *HM* and *∆Certainty* are included in the model (see Table 7, column 3). This suggests an overall increase in tax risk over time independent of HM participation, because participants appeared to perceive a slightly larger increase of tax risk when they assessed change for a greater number of years.

**Table 7:** Model coefficients of the mediation effect of *HM* on *∆TaxRisk* via *∆Certainty*

	Dependent variables		
Variables	(1) *∆TaxRisk*	(2) *∆Certainty*	(3) *∆TaxRisk*
**Direct effects**			
*HM*	−1.51** (0.60)	2.67*** (0.70)	−0.63 (0.65)
*∆Certainty*	–	–	−0.33** (0.13)
*Years*	0.16 (0.10)	0.02 (0.12)	0.17* (0.10)
*Constant*	−0.17 (0.92)	−0.56 (1.09)	−0.35 (0.86)
*R*^*2*^	0.32	0.34	0.43
**Indirect effects**			
*HM* via *∆Certainty*	–	–	−0.88** (0.44)
**Total effects**			
*HM*	−1.51** (0.60)	2.67*** (0.70)	−1.51** (0.60)

Coefficients and standard errors (in parentheses) were estimated using maximum likelihood estimation with robust standard errors. The dummy variable *HM* represents HM participation, *∆Certainty* and *∆TaxRisk* reflect perceived changes in tax certainty and tax risk, respectively. *Years* represents the timeframe for which participants were asked to indicate change. **Direct effects** correspond to parameter estimates in multiple regressions and represent the effects net of any effect by control variables included in the model. **Indirect effects** of *HM* represent the mediation effect and equals the product of the direct effects of *HM* on *∆Certainty* (Column 2) and of *∆Certainty* on *∆TaxRisk* (Column 3). **Total effects** of HM represent the sum of direct and indirect effects and corresponds to the coefficient of *HM* in a regression on *∆TaxRisk* without controlling for *∆Certainty* (Column 1). ***, **, and * indicate two-tailed statistical significance at the 1, 5, and 10% level, respectively.

The next model (see Table 8 for model coefficients) reveals a significant total effect of *HM* on *CurrTaxRisk* of −1.175, indicating that HM firms report lower values of perceived current tax risk than the control group. However, we do not find support for hypothesis H2a, because the difference in perceived current tax risk is not significantly mediated by the perceived change in tax risk (*∆TaxRisk*), owing to the lack of association between *∆TaxRisk* and *CurrTaxRisk.* These results leave room for alternative explanations of the difference in *CurrTaxRisk* than the improvements experienced during HM participation. In particular, differences in tax risk may have existed before HM (non-)participation, which suggests that tax risk has influenced the self-selection or acceptance into the HM program.

**Table 8:** Model coefficients of the mediation effect of *HM* on *CurrTaxRisk* via *∆TaxRisk*

	Dependent variables		
Variables	(1) *CurrTaxRisk*	(2) *∆TaxRisk*	(3) *CurrTaxRisk*
**Direct effects**			
*HM*	−1.19*** (0.35)	−1.51** (0.60)	−1.11*** (0.42)
*∆TaxRisk*	–	–	0.04 (0.16)
*Years*	–	0.16 (0.11)	–
*Constant*	3.19*** (0.21)	−0.16 (0.97)	3.15*** (0.28)
*R*^*2*^	0.17	0.32	0.17
**Indirect effects**			
*HM* via *∆TaxRisk*	–	–	−0.06 (0.25)
**Total effects**			
*HM*	−1.19*** (0.35)	−1.51** (0.60)	−1.18** (0.34)

Coefficients and standard errors (in parentheses) were estimated using maximum likelihood estimation with robust standard errors. The dummy variable *HM* represents HM participation, and *∆TaxRisk* reflect perceived changes in tax risk. *CurrTaxRisk* is the perceived current tax risk. *Years* represents the timeframe for which participants were asked to indicate change. **Direct effects** correspond to parameter estimates in multiple regressions and represent the effects net of any effect by control variables included in the model. **Indirect effects** of *HM* represent the mediation effect and equals the product of the direct effects of *HM* on *∆TaxRisk* (Column 2) and of *∆TaxRisk* on* CurrTaxRisk* (Column 3). **Total effects** of HM represent the sum of direct and indirect effects and corresponds to the coefficient of *HM* in a regression on *CurrTaxRisk* without controlling for *∆TaxRisk* (Column 1). ***, **, and * indicate two-tailed statistical significance at the 1, 5, and 10% level, respectively.

With regard to H3, we again find that HM firms report significantly higher perceived current tax risk management quality, as expressed by a significant total effect of *HM* on *CurrTRM* of 1.121 (see Table 9 for model coefficients). Concerning H4, we again do not find any evidence that firms experienced different developments in tax risk management quality during HM participation. Regarding H4a, we do not find a mediated effect of *HM* on *CurrTRM* due to the very similar responses in *∆TRM*. These results also invite other explanations for the difference in *CurrTRM* than the improvements perceived during HM participation. As with tax risk, differences in TRM could thus have existed before HM (non-)participation, possibly leading to a higher likelihood of HM participation for firms with more advanced TRM.

**Table 9:** Model coefficients of the mediation effect of HM on *CurrTRM* via *∆TRM*

	Dependent variables		
Variables	(1) *CurrTRM*	(2) *∆TRM*	(3) *CurrTRM*
**Direct effects**			
*HM*	1.23*** (0.36)	−0.03 (0.34)	1.15*** (0.40)
*∆TRM*	–	–	0.95*** (0.25)
*Years*	–	−0.04 (0.06)	–
*Constant*	4.25*** (0.28)	1.48*** (0.56)	3.17*** (0.43)
*R*^*2*^	0.11	0.01	0.37
**Indirect effects**			
*HM* via *∆TRM*	–	–	−0.03 (0.32)
**Total effects**			
*HM*	1.23*** (0.36)	−0.03 (0.34)	1.12*** (0.41)

Coefficients and standard errors (in parentheses) were estimated using maximum likelihood estimation with robust standard errors. The dummy variable *HM* represents HM participation, and *∆TRM* reflect perceived changes in the quality of tax risk management. *CurrTRM* is the perceived current quality of tax risk management. *Years* represents the timeframe for which participants were asked to indicate change. **Direct effects** correspond to parameter estimates in multiple regressions and represent the effects net of any effect by control variables included in the model. **Indirect effects** of *HM* represent the mediation effect and equals the product of the direct effects of *HM* on *∆TRM* (Column 2) and of *∆TRM* on* CurrTRM* (Column 3). **Total effects** of HM represent the sum of direct and indirect effects and corresponds to the coefficient of *HM* in a regression on *CurrTRM* without controlling for *∆TRM* (Column 1). ***, **, and * indicate two-tailed statistical significance at the 1, 5, and 10% level, respectively.

**Fig. 4:**
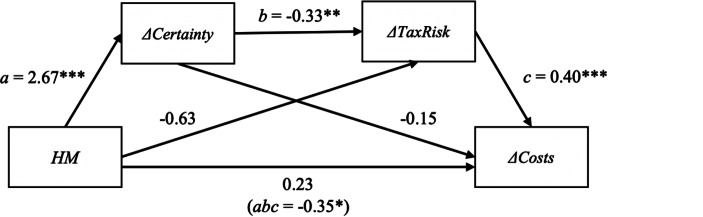
Illustration of the mediation model displayed in Table 10. Coefficients represent direct effects and the indirect mediation effect in parentheses. Effects on *∆Certainty, ∆TaxRisk* and *∆Costs* are controlled for *Years* (not depicted)*.* ***, **, and * indicate two-tailed statistical significance at the 1, 5, and 10% level, respectively.

Further analyzing the difference in the change in compliance costs experienced by firms (*∆Costs*) and its potential mediators, we find a non-significant difference in the perceived change of compliance costs (*∆Costs*) of −0.777. However, we still detect significant mediations via *∆Certainty* (H5a) as well as *∆TaxRisk* (H5b), but not via *∆TRM* (H5c). With a simultaneous analysis of all three mediators, indirect effects add up to a total of −1.000, which exceeds the total effect. We thus find support for hypotheses H5a and H5b. Further exploring the relationship between *HM, ∆*Certainty, *∆TaxRisk*, and *∆Costs,* we find a dual mediation (significant at the 10% level) between the four variables while controlling other simple indirect and direct effects (see Fig. 4 and Table 10, Column 6).

**Table 10:** Model coefficients of the effects of *HM* on *∆Costs*

	Dependent variables					
Variables	(1) *∆Costs*	(2) *∆Costs*	(3) *∆Costs*	(4) *∆Costs*	(5) *∆Costs*	(6) *∆Costs*
**Direct effects**						
*HM*	−0.78 (0.50)	0.02 (0.59)	−0.07 (0.52)	−0.77 (0.50)	0.22 (0.53)	0.23 (0.53)
*∆Certainty*	–	−0.28** (0.12)	–	–	−0.16 (0.11)	−0.15 (0.11)
*∆TaxRisk*	–	–	0.47*** (0.15)	–	0.39** (0.17)	0.40*** (0.15)
*∆TRM*	–	–	–	0.28 (0.19)	0.05 (0.24)	–
*Years*	0.10 (0.12)	0.11 (0.11)	0.03 (0.09)	0.11 (0.11)	0.05 (0.08)	0.04 (0.08)
*Constant*	0.43 (1.08)	0.27 (0.99)	0.51 (0.79)	0.01 (1.03)	0.33 (0.72)	0.41 (0.76)
*R*^*2*^	0.14	0.24	0.35	0.17	0.33	0.37
**Indirect effects**						
*HM* via *∆Certainty*	–	−0.76** (0.32)	–	–	−0.41 (0.30)	−0.40 (0.28)
*HM* via *∆TaxRisk*	–	–	−0.71** (0.29)	–	−0.59** (0.27)	−0.25 (0.28)
*HM* via *∆TRM*	–	–	–	−0.007 (0.097)	−0.001 (0.019)	–
*HM* via *∆Certainty* via* ∆TaxRisk*	–	–	–	–	–	−0.353* (0.187)
*HM* (total indirect)	–	–	–	–	−1.000*** (0.333)	−1.008*** (0.343)
**Total effects**						
*HM*	−0.777 (0.501)	−0.777 (0.501)	−0.777 (0.501)	−0.777 (0.501)	−0.777 (0.501)	−0.777 (0.501)

Direct effects of *HM* on *∆Certainty* and *∆TaxRisk*, and on *∆TRM* are displayed in Tables 7 and 9, respectively. Coefficients and standard errors (in parentheses) were estimated using maximum likelihood estimation with robust standard errors. The dummy variable *HM* represents HM participation. *∆Costs* is the perceived change in compliance costs. *∆Certainty* and *∆TaxRisk* reflect perceived changes in tax certainty and tax risk, respectively. *∆TRM* represents the perceived changes in the quality of tax risk management. *Years* represents the timeframe for which participants were asked to indicate change. **Direct effects** correspond to parameter estimates in multiple regressions and represent the effects net of any effect by control variables included in the model. **Indirect effects** displayed in Column 2–5 of *HM* represent the simple mediation effects and equal the product of the direct effects of *HM* on the mediator (see Tables 7 and 9) and of the mediator on* ∆Costs* (Column 2–5). Indirect effects displayed in Column 6 include the coefficients of a dual mediation, which is the product of the effect of *HM* on *∆Certainty*, of *∆Certainty* on *∆TaxRisk*, and of *∆TaxRisk* on *∆Costs.* **Total effects** of HM represent the sum of the direct and indirect effects and corresponds to the coefficient of *HM* in a regression on *∆Costs* without controlling for any mediator (Column 1). ***, **, and * indicate two-tailed statistical significance at the 1, 5, and 10% level, respectively.

#### Robustness Tests

In addition to the prior analyses using U‑Tests and Spearman correlations, we further ensure that results are not excessively driven by single outliers. To this end, we use jackknife resampling, which is based on consecutively dropping each observation from the sample, producing more conservative standard errors than bootstrapping and thus reducing significance of some effects. However, all previously significant effects remain significant at the 10% level (see Table A.8 in Appendix A).

Moreover—similar to jackknife resampling—we repeatedly compute all original models with each participant being dropped once from the sample, resulting in a total of 40 estimates for each model parameter. As expected, dropping the most influential cases decreases the size and significance of all coefficients. However, all previously significant findings remain significant at least at the 10% level, except the twofold mediation effect of HM on *∆Costs* (see Table A.8 in Appendix A).

### Additional Analyses

To provide deeper insights into differences between HM firms and the control group, we assess responses to a wide range of other items and scales used in the questionnaire. The following subsections outline perceived changes in tax compliance and the relationship with tax authorities, in perceived changes of specific types of tax risk and tax risk sources, in current sources of tax risk and tax risk management methods, in expectations from HM, and in attitudes towards tax compliance and risk. Because some questions appeared earlier in the questionnaire than questions addressing our main constructs of interest, we collected more responses to some items than for our main analyses. Descriptive statistics and group comparisons of items covered in this section are presented in Appendix A.

#### Reported Changes in Tax Compliance and the Relationship with Tax Authorities

As two main goals of cooperative compliance are to improve the relationship between taxpayers and tax authorities and to foster tax compliance in the long run, we inquired about the perceived change of these two potential HM outcomes. On average, both groups report a very similar increase in self-reported tax compliance. HM firms, however, report a considerable and significantly stronger improvement in the relationship quality than the control group (see Table A.1 in Appendix A).

#### Perceived Changes in Specific Types of Tax Risk and Tax Risk Sources

We also inquired on the change of more specific types of tax risk, namely the risk of penalties for the firm and for individual decision makers, the risk of litigation, the own personal risk for the participant, and reputational risk for the company. We find the same pattern as for general tax risk in responses to these items: the control group indicated significant increases in risk, while HM firms indicated slight decreases (see Table A.2 in Appendix A).

In line with Mulligan and Oats (2009) and Wunder (2009), the survey also inquired on seven specific risk sources (transactional, operational, compliance, financial accounting, portfolio, management, reputational) and how participants perceived their change since entering HM or during the past years. Results mostly reflect the patterns found in other items measuring change in risk, particularly for transactional risk, operational risk, compliance risk, and management risk (see Table A.2 in Appendix A).

#### Sources of Tax Risk

In addition to the general measure of current tax risk used in the main analysis, again based on the classification of tax risk sources based on Mulligan and Oats (2009) and Wunder (2009), our survey also covered the specific risk sources (transactional, operational, compliance, financial accounting, portfolio, management, reputational) at the present time. While the control group indicated a significantly lower current operational risk, it also perceived a significantly higher compliance risk and management risk (see Table A.3 in Appendix A). While these differences could indicate underlying differences in the risk profile of the two groups, they may also reflect positive effects of HM, particularly on compliance risk.

#### Current Tax Risk Management Methods

The survey also assessed the importance of seven distinct tax risk management methods, i.e., “systems to and/or procedures to identify and manage tax risk” in the company. The items covered: advance rulings by the tax administration, advance informal agreements with the tax administration, external advisors, extensive documentation, cost analysis on possible financial penalties, smell test based on individual experience and judgment, and following a benchmark firm.

We find similar responses by the two groups in most items, expect for informal agreements and external tax advisors, with participants of HM firms reporting utilization of more informal agreements and less external advisors than the control group (see Table A.4 in Appendix A). These differences may reflect a stronger reliance on feedback and agreements with the authorities and less reliance on external tax advisors due to HM.

#### Expectations About Horizontal Monitoring and Importance of Goals

Before participants answered questions about the actual changes their firms had experienced, we asked them to indicate what changes they would expect (or, in the case of HM firms, had expected before entering HM) from participation in the HM program and how important they consider these possible changes. Items reflect the same topics covered in the main analysis, including tax risk, tax certainty, and compliance costs. For instance, we asked if participants expected an increase or a decrease in tax risk due to HM participation (on a scale from −3 to +3), and, how important they consider the goal of reducing tax risk (on a scale from 1 to 7). All items measuring expectations and perceived importance were only displayed if participants indicated that they had at least heard of HM, which reduced the number of participants in the control group. Descriptive statistics and group comparisons of expectations and the perceived importance are reported in Tables A.5 and A.6 in Appendix A.

Overall, expectations about the HM projects appear to be similarly positive in the two groups, with both groups expressing particularly high importance of increasing tax certainty as well as high expectations about an increase of tax certainty through HM. Similarly, both groups attributed high importance to reducing tax risk and expected this goal to be achieved by the HM program. Only regarding items covering the risk of penalties, we find significant differences, with the control group expecting a stronger reduction in these risks. Concerning the importance of goals, we find no significant differences between the two groups. Overall, these results indicate mostly similar expectations regarding HM performance and similar priorities regarding potential improvements.

#### Attitudes Towards Tax Compliance and Risk

To assess differences in participants’ and firms’ understanding of tax compliance, we asked participants how much they personally—and their firm, respectively—would agree that tax compliance is a matter of “following the letter of the law”, and of “following the spirit of the law”. Moreover, participants were asked to indicate their personal risk attitude. Overall, participants from HM firms and non-HM firms show a similar attitude towards tax compliance and risk. However, we do find that HM firms indicate a (marginally significantly) higher importance of following the letter of the law than the control group (see Table A.7 in Appendix A). This may suggest that HM firms give slightly more priority to tax compliance than the control group.

## Discussion and Conclusion

The way in which tax administrations and firms interact affects the governance structure and risk profile of firms. When the relationship between management and tax administrations is viewed from a principal-agent perspective, the state assumes the principal’s role that participates in firms’ profits by claiming a share via taxes. As a result, states and tax authorities have an inherent interest in reducing information asymmetries.

We propose that high-quality tax risk management and transparency obligations commonly found in CC programs are alternative measures to reduce information asymmetries. In CC, firms provide transparency and establish tax control frameworks as part of their tax risk management systems. Tax administrations, on the other hand, discuss with firms the appropriate tax treatment of (complex) transactions at an early stage, thus offering tax certainty. Therefore, CC may reduce agency and signaling costs compared to conventional ex-post audits and may thus offer benefits for firms and tax administrations alike. Against this theoretical background, we use the case of the Austrian HM pilot project for a survey study. We analyze the association of HM participation with tax risk, with the quality of tax risk management systems, and with compliance costs.

We find strong evidence that HM firms experienced decreases in tax risk and compliance costs—differences that appear to be mediated by an increase in tax certainty. However, surprisingly, the perceived change in tax risk appears to be only weakly associated with the perceived current tax risk. These results may indicate that firms’ current tax risk is determined predominantly by unobserved factors and not by the changes reported by participants. While this might point to a higher likelihood of low-tax-risk firms applying and being accepted for the HM pilot project, it could also be due to other reasons, such as biased perceptions by participants. Results also indicate, in line with expectations, that HM firms already had more advanced tax risk management before participation in the HM pilot project: HM firms report significantly better current tax risk management, but a similar rate of tax risk management improvement as the control group. The significant association between the perceived current quality in tax risk management and its perceived change further support this conclusion.

Overall, we find that HM firms perceive distinct benefits from the pilot project. Our results mostly support the notion that CC is an effective measure to reduce information asymmetries between principal and agent, as well as costs for the firm. Significantly, our findings suggest that CC reduces tax risk and compliance costs predominantly by increasing tax certainty. We also find some evidence that firms with better tax risk management and lower tax risk are more likely to participate in HM. This supports the idea that CC is more suitable for firms with overall less risky tax strategies because the benefits of CC should outweigh the potential benefits of more risky tax planning. These results align with our expectations and the notion of responsive regulation, which suggests that cooperative and service-oriented policies should be targeted at inherently compliant taxpayers.

Despite the small number of HM firms, we find significant associations between HM and some variables of interest. Large differences in the perceived change in tax risk and tax certainty are especially noteworthy. To ensure that results are not driven by outliers, we also use non-parametric U‑tests and Pearson correlations, as well as repeated computations of our mediation models in which each observation is dropped once to provide a “worst-case” estimate of coefficients when the most influential observation is dropped. Our results hold.

We find additional insights into the differences between HM firms and the control group and further support for our results in an additional exploration of responses: The perceived reduction in tax risk by HM firms is also reflected in more specific types of risk, such as litigation risk or reputational risk. While HM firms do not report a stronger increase in tax compliance, they perceive that their relationship with tax authorities has improved significantly and that they rely more on informal advance agreements with tax authorities as a method of tax risk management than the control group.

Our study is subject to several limitations. First, despite our efforts to disentangle reasons for and consequences of HM participation, our sample for both the treatment group and control group is subject to (self-)selection bias. Moreover, the small sample size may limit the generalizability of our results. Nevertheless, differences in many variables are unambiguous, and our sample covers the majority of Austrian HM firms. Second, the cross-sectional study design prohibits conclusive interpretation regarding causality. However, by using items about perceived change as well as the perceived current state, we were able to shed more light on possible causes and effects of CC. Third, our results are based on subjective assessments and voluntary participation in the survey. Differences between groups may thus be subject to biased perceptions, particularly confirmation bias, i.e., the tendency to justify the decision to participate in HM by overestimating its benefits. However, our participants’ expert status speaks in favor of the validity and relevance of our results, as most participants are high-ranking employees and thus likely to be deeply involved in tax decisions of the firms. Fourth, our analysis is limited to large Austrian firms with a turnover of more than 40 million Euro, which have a probability of a conventional tax audit of nearly 100%. However, the Austrian HM project can be considered a prototypical CC program that closely follows the principles laid out by the OECD. Several countries limit HM to large businesses to match costs and benefits for tax authorities. Furthermore, Austria shows many similarities with other countries, particularly Germany, with regard to the tax system and macroeconomic, cultural, and legal features (e.g., Hoppe et al. 2019). We thus expect our results to be informative for other HM initiatives around the globe.

Our study is the first to examine tax risk and tax risk management as both possible reasons for and consequences of CC participation from firms’ perspective. While our research design does not allow direct identification of causality, we analyze HM firms and a control group with regard to both the perceived current state and the perceived changes in our variables of interest. In conjunction with mediation analyses, our approach provides additional information about the causes and effects of HM participation.

As CC is a relatively young concept, early analysis of its effects is valuable and important, and future research based on objective data is needed to corroborate our results in other countries. Our findings underscore the importance and promise of cooperative relationships that reduce costly information asymmetries and provide increased certainty for both sides. Therefore, we expect our results to be of interest to policymakers and firms alike, regarding both the decision to participate in a CC program and the design of cooperative tax policies.

## Appendix A: Tables from additional analyses

**Table A.1:** Descriptive statistics and group comparisons of changes in tax compliance and in the relationship with tax authorities

	HM firms (*n* = 9)				Control group (*n* = 31)				Comparisons	
Variables	Mean	*SD*	Median	Mean rank	Mean	*SD*	Median	Mean rank	Mean difference	Mean rank difference
*Perceived changes (strong decrease (−3)–strong increase (+3))*										
Compliance with tax laws	0.78	1.30	0	20.39	0.65^+++^	0.98	0	20.53	0.13	−0.14
Relationship quality with tax authorities	1.89^+++^	1.27	2	30.11	0.16	1.39	0	17.71	1.73***	12.40***

Significance levels are based on conventional independent t‑Tests (for mean differences) and on exact U‑tests (for mean rank differences). ***, ** and * indicate two-tailed statistical significance at the 1, 5 and 10% level, respectively. ^+^, ^++^, and ^+++^ denote means of items/scales measuring perceived change which are significantly different from zero at the 1, 5, and 10% level, respectively.

**Table A.2:** Descriptive statistics and group comparisons of changes in specific types of tax risk and risk sources

	HM firms (*n* = 9)				Control group (*n* = 31)				Comparisons	
Variables	Mean	*SD*	Median	Mean rank	Mean	*SD*	Median	Mean rank	Mean difference	Mean rank difference
*Perceived changes in specific tax risks (strong decrease (−3)–strong increase (+3))*										
Risk of penalties	−1.11^+^	1.45	0	9.00	1.03^+++^	1.22	1	23.84	−2.14***	−14.84***
Risk of litigation	−0.89^++^	1.05	−1	8.67	0.94^+++^	1.15	1	23.94	−1.82***	−15.27***
Risk of penalties for individual decision makers	−0.78	1.30	0	10.83	0.74^+++^	1.13	1	23.31	−1.52***	−12.47***
Personal risk	−0.56	1.59	0	14.00	0.55^+++^	1.09	0	22.39	−1.10**	−8.39**
Reputational risk	−0.22	1.39	0	13.61	0.87^+++^	1.31	1	22.50	−1.09**	−8.89**
*Changes in tax risk sources (strong decrease (−3)–strong increase (+3))*										
Transactional risk (examples: acquisitions, mergers)	−0.44	1.67	0	9.00	0.74^+++^	1.18	1	22.90	−1.17**	−13.90***
Operational risk (examples: new business ventures, new operating models, new operating structure)	−0.78	1.39	−1	8.67	1.23^+++^	1.26	1	23.69	−2.00***	−15.03***
Compliance risk (examples: weak records and controls, legislative changes)	−0.67	1.66	−1	10.83	0.68^++^	1.45	1	22.86	−1.34**	−12.02**
Financial accounting risk (examples: changes in systems and policies)	0.00	0.87	0	14.00	0.32^++^	1.19	0	21.57	−0.32	−7.57
Management risk (examples: changes in personnel, new/inexperienced resources)	−0.11	0.33	0	13.61	0.35^++^	0.92	0	22.19	−0.47	−8.58*
Reputational risk (example: revenue authority investigation)	0.00	1.23	0	9.00	0.55^++^	1.31	0	21.77	−0.55	−12.77
Portfolio risk (example: combination of any of the risks)	−0.11	0.93	0	8.67	0.58^++^	1.26	1	22.45	−0.69	−13.79**

Significance levels are based on conventional independent t‑Tests (for mean differences) and on exact U‑tests (for mean rank differences). ***, ** and * indicate two-tailed statistical significance at the 1, 5 and 10% level, respectively. ^+^, ^++^, and ^+++^ denote means of items/scales measuring perceived change which are significantly different from zero at the 1, 5, and 10% level, respectively.

**Table A.3:** Descriptive statistics and group comparisons of current sources of tax risk

	HM firms (*n* = 11)				Control group (*n* = 36)				Comparisons	
Variables	Mean	*SD*	Median	Mean rank	Mean	*SD*	Median	Mean rank	Mean difference	Mean rank difference
*Sources of tax risk (not at all (1)–to a great extent (7))*										
Transactional risk (examples: acquisitions, mergers)	3.91	2.07	3	23.86	4.00	1.85	4	24.04	−0.09	−0.18
Operational risk (examples: new business ventures, new operating models, new operating structure)	4.82	1.60	5	30.27	3.78	1.77	4	22.08	1.04*	8.19*
Compliance risk (examples: weak records and controls, legislative changes)	2.64	1.03	2	15.32	3.81	1.45	4	26.65	−1.17**	−11.33**
Financial accounting risk (examples: changes in systems and policies)	2.64	1.12	2	18.50	3.39	1.48	3	25.68	−0.75	−7.18
Management risk (examples: changes in personnel, new/inexperienced resources)	3.00	1.48	3	17.82	3.83	1.54	4	25.89	−0.83	−8.07*
Reputational risk (example: revenue authority investigation)	2.73	2.20	2	19.00	3.53	1.81	3.5	25.53	−0.80	−6.53
Portfolio risk (example: combination of any of the risks)	3.27	1.62	3	20.86	3.72	1.63	3.5	24.96	−0.45	−4.10

Significance levels are based on conventional independent t‑Tests (for mean differences) and on exact U‑tests (for mean rank differences). ***, ** and * indicate two-tailed statistical significance at the 1, 5 and 10% level, respectively.

**Table A.4:** Descriptive statistics and group comparisons of the importance of tax risk management methods

	HM firms (*n* = 11)				Control group (*n* = 36)				Comparisons	
Variables	Mean	*SD*	Median	Mean rank	Mean	*SD*	Median	Mean rank	Mean difference	Mean rank difference
*Importance of tax risk management methods (not important at all (1)–very important (7))*										
Binding advance rulings from tax administration	3.73	2.05	4	24.50	3.58	2.12	3	23.85	0.14	0.65
Advance informal agreements with tax administration	5.00	2.19	6	31.18	3.56	1.99	3	21.81	1.44**	9.37**
External advisors	5.18	1.40	5	17.91	5.97	1.08	6	25.86	−0.79*	−7.95*
Extensive documentation	5.91	1.22	6	26.32	5.47	1.59	6	23.29	0.44	3.03
Cost analysis of potential penalties	2.55	2.02	2	21.91	2.69	1.72	2	24.64	−0.15	−2.73
“Smell test” based on individual experience and judgement	3.00	1.79	3	20.68	3.56	1.75	3	25.01	−0.56	−4.33
Follow a benchmark firm	3.00	1.84	2	20.23	3.64	1.87	3	25.15	−0.64	−4.92

Significance levels are based on conventional independent t‑Tests (for mean differences) and on exact U‑tests (for mean rank differences). ***, ** and * indicate two-tailed statistical significance at the 1, 5 and 10% level, respectively.

**Table A.5:** Descriptive statistics and group comparisons for expectations on Horizontal Monitoring

	HM firms (*n* = 10)				Control group (*n* = 28)				Comparisons	
Variables	Mean	*SD*	Median	Mean rank	Mean	*SD*	Median	Mean rank	Mean difference	Mean rank difference
*Expectations on the effects of Horizontal Monitoring (strong decrease (−3)–strong increase (+3))*										
Tax certainty	1.90^+++^	1.66	2.5	21.15	1.82^+++^	1.39	2	18.91	0.08	2.24
Compliance costs	0.20	0.79	0	17.50	0.29	1.18	0.5	20.21	−0.09	−2.71
Risk of penalties	−0.20	1.40	0	24.85	−1.11^+++^	1.29	−1	17.59	0.91*	7.26*
Risk of penalties for individuals	−0.30	1.16	0	24.45	−1.11^+++^	1.20	−1	17.73	0.81*	6.72*
Tax risk	−1.30^++^	1.70	−2	17.60	−1.04^+++^	1.40	−1	20.18	−0.26	−2.58
Personal risk	−0.30	1.06	0	22.15	−0.64^+++^	1.10	−1	18.55	0.34	3.60
Reputational risk	−0.40	1.17	0	21.55	−0.75^+++^	1.38	−1	18.77	0.35	2.78
Risk of litigation	−0.50	1.35	−0.5	19.95	−0.61^++^	1.34	−1	19.34	0.11	0.61
Compliance with tax laws	0.10	0.32	0	18.60	0.18	0.98	0	19.82	−0.08	−1.22
Relationship quality with tax authorities	1.10^++^	1.29	0.5	18.05	1.21^+++^	0.99	1	20.02	−0.11	−1.97

This table displays means, medians, and mean ranks of items measuring sources of tax risk as well as group comparison of means and mean ranks. Significance levels are based on conventional independent t‑Tests (for mean differences) and on exact U‑tests (for mean rank differences). ***, ** and * indicate two-tailed statistical significance at the 1, 5 and 10% level, respectively. ^+^, ^++^, and ^+++^ denote means of items/scales measuring perceived change which are significantly different from zero at the 1, 5, and 10% level, respectively.

**Table A.6:** Descriptive statistics and group comparisons for the perceived importance of goals

	HM firms (*n* = 10)				Control group (*n* = 32)				Comparisons	
Variables	Mean	*SD*	Median	Mean rank	Mean	*SD*	Median	Mean rank	Mean difference	Mean rank difference
*Importance of goals (not important at all (1) very important (7))*										
Increase tax certainty	6.50	0.71	7	24.95	6.03	1.18	6	20.42	0.47	4.53
Reduce compliance costs	4.80	1.40	5	21.60	4.69	1.77	5	21.47	0.11	0.13
Reduce risk of penalties	5.00	2.00	5.5	19.20	5.44	1.83	6	22.22	−0.44	−3.02
Reduce risk of penalties for individuals	4.40	1.96	4.5	16.35	5.34	1.91	6	23.11	−0.94	−6.76
Reduce tax risk	5.30	1.49	5.5	19.85	5.56	1.22	6	22.02	−0.26	−2.17
Reduce personal risk	4.30	1.89	4.5	18.60	4.91	1.75	5	22.41	−0.61	−3.81
Reduce reputational risk	4.40	1.71	4.5	16.10	5.34	1.64	6	23.19	−0.94	−7.09
Reduce risk of litigation	4.70	1.83	5	21.05	4.78	1.64	5	21.64	−0.08	−0.59
Increase compliance with tax laws	5.30	1.57	6	26.60	4.41	1.70	4.5	19.91	0.89	6.69
Improve relationship quality with tax authorities	5.70	1.25	5.5	23.75	5.25	1.48	5.5	20.8	0.45	2.95

This table displays means, medians, and mean ranks of items measuring sources of tax risk as well as group comparison of means and mean ranks. Significance levels are based on conventional independent t‑Tests (for mean differences) and on exact U‑tests (for mean rank differences). ***, ** and * indicate two-tailed statistical significance at the 1, 5 and 10% level, respectively.

**Table A.7:** Descriptive statistics and group comparisons of attitudes towards tax compliance and risk

	HM firms (*n* = 9)				Control group (*n* = 31)				Comparisons	
Variables	Mean	*SD*	Median	Mean rank	Mean	*SD*	Median	Mean rank	Mean difference	Mean rank difference
*“In my company, tax compliance means following the …” (strongly disagree (1)–strongly agree (7))*										
Letter of tax laws	5.78	0.97	6	25.89	4.81	1.60	5	18.94	0.97*	6.95
Spirit of tax laws	5.89	1.90	6	23.00	5.87	1.12	6	19.77	0.02	3.23
*“To me personally, tax compliance means following the …” (strongly disagree (1)–strongly agree (7))*										
Letter of tax laws	5.67	0.87	6	26.06	4.77	1.54	5	18.89	0.89	7.17*
Spirit of tax laws	5.89	1.90	6	19.89	6.16	1.13	7	20.68	−0.27	−0.79
*Personal risk attitude (very risk-averse (1)–very risk-seeking (7))*										
Risk attitude	3.00	1.32	3	16.44	3.61	1.23	4	21.68	−0.61	−5.24

Significance levels are based on conventional independent t‑Tests (for mean differences) and on exact U‑tests (for mean rank differences). ***, ** and * indicate two-tailed statistical significance at the 1, 5 and 10% level, respectively.

**Table A.8:** Coefficients and their respective *p*-values from models with dropped observations

	Original sample (*N* = 40)			Dropped observations (*N* − 1)					
Coefficients	*B*	*p*_*rob*_	*p*_*jn*_	*B*_*mean*_	*B*_*min*_	*B*_*max*_	*p*_*mean*_	*p*_*min*_	*p*_*max*_
*Effects of mediation model: HM on ∆TaxRisk via ∆Certainty* (Table 7)									
*HM on ∆TaxRisk* (total effect)	−1.513	0.012	0.029	−1.513	−1.213	−1.790	0.014	0.001	0.028
*HM* on* ∆TaxRisk* (direct effect)	−0.633	0.354	0.418	−0.633	−0.300	−0.995	0.367	0.138	0.639
*Certainty on ∆TaxRisk*	−0.330	0.026	0.057	−0.330	−0.272	−0.446	0.030	<0.001	0.063
*HM on ∆Certainty*	2.666	<0.001	<0.001	2.666	2.503	2.925	<0.001	<0.001	<0.001
*HM on ∆TaxRisk via ∆Certainty* (indirect effect)	−0.880	0.033	0.065	−0.880	−0.760	−1.201	0.037	0.002	0.078
*Effects of mediation model: HM on CurrTaxRisk via ∆TaxRisk* (Table 8)									
*HM* on *CurrTaxRisk* (total effect)	−1.175	<0.001	0.003	−1.175	−1.044	−1.393	<0.001	<0.001	0.002
*HM* on* CurrTaxRisk* (direct effect)	−1.114	0.008	0.022	−1.114	−1.114	−1.239	0.010	0.002	0.033
*∆TaxRisk* on* CurrTaxRisk*	0.040	0.806	0.183	0.041	−0.008	0.147	0.802	0.360	0.990
*HM* on *∆TaxRisk*	−1.513	0.012	0.027	−1.513	−1.213	−1.790	0.014	0.001	0.028
*HM* on *CurrTaxRisk* via *∆TaxRisk* *(indirect effect)*	−0.061	0.810	0.831	−0.062	0.012	−0.263	0.808	0.368	0.990
*Effects of mediation model: HM on CurrTRM via ∆TRM* (Table 9)									
*HM* on *CurrTRM* (total effect)	1.121	0.006	0.016	1.120	0.943	1.305	0.007	0.001	0.014
*∆TRM* on* CurrTRM*	0.952	<0.001	0.001	0.952	0.846	1.073	<0.001	<0.001	0.004
*HM* on *∆TRM*	−0.026	0.939	0.945	−0.026	0.133	−0.205	0.893	0.528	0.997
*HM* on *CurrTRM* via *∆TRM*	−0.025	0.939	0.945	−0.025	−0.025	−0.200	0.893	0.538	0.997
*Effects of mediation model: HM on ∆Costs via ∆Certainty, ∆TaxRisk, and ∆TRM* (Table 10, column 5)									
*HM* on *∆Costs* (total)	−0.78	0.12	0.20	−0.78	−0.56	−1.09	0.13	0.01	0.26
*HM* on *∆*Costs (direct)	0.22	0.53	0.73	0.22	−0.16	0.45	0.67	0.37	0.93
*HM on ∆Costs* via *∆TaxRisk*	−0.59	0.03	0.06	−0.58	−0.48	−0.72	0.03	0.01	0.08
*HM* on *∆Costs* *(total indirect)*	−1.00	0.00	0.02	−1.00	−0.89	−1.22	0.00	0.00	0.01
*HM* via *∆Certainty* via *∆TaxRisk* (column 6)	−0.35	0.06	0.10	−0.35	−0.27	−0.45	0.07	0.05	0.11

This table shows robustness checks for the results of mediation models reported in Tables 7, 8, 9 and 10. In the columns under *Original sample* the table shows original regression coefficients (*B*) and *p*-values based on robust standard errors (*p*_*rob*_, original models were already computed using robust standard errors), including *p*-values based on the jackknife resampling method (*p*_*jn*_). Jackknife resampling resembles bootstrapping and is based on dropping each observation once, thus repeatedly estimating the coefficients of interest. In addition, we repeatedly compute models with each observation being dropped once from the sample. This results in 40 estimates of parameters and *p*-values, of which we report means, minima, and maxima under *Dropped observations*. *B*_*min*_ and *p*_*max*_ thus reflect the smallest and least significant absolute effect found by dropping the observation (or one of the observations) which contribute most to the original estimate.

## Appendix B: Full questionnaire

**Table B.1:** Full questionnaire

English version	German version
**Familiarity with Horizontal Monitoring**	
How familiar are you with horizontal monitoring in Austria?	Wie vertraut sind Sie mit Horizontal Monitoring in Österreich?
*Answer options*	
□ Good knowledge	□ Gute Kenntnisse
□ Somewhat familiar	□ Etwas vertraut
□ Heard of it	□ Habe davon gehört
□ Not at all familiar	□ Überhaupt nicht vertraut
**Sources of Tax Risk (present)**	
What are the sources of tax risk in your company?	Welche Quellen hat steuerliches Risiko in Ihrem Unternehmen?
*Items*	
Transactional risk (examples: acquisitions, mergers)	Transaktionsrisiko (z. B.: Akquisitionen, Verschmelzungen)
Operational risk (examples: new business ventures, new operating models, new operating structure)	Operatives Risiko (z. B.: neue Geschäftsfelder, neue Prozesse, neue Strukturen)
Compliance risk (examples: weak records and controls, legislative changes)	Compliance-Risiko (z. B.: Schwächen bei Aufzeichnungen oder Kontrollen, gesetzliche Änderungen)
Financial accounting risk (examples: changes in systems and policies)	Rechnungslegungsrisiko (z. B.: Änderungen in Rechnungslegungsprozessen oder -politik)
Management risk (examples: changes in personnel, new/inexperienced resources)	Management-Risiko (z. B.: Personalwechsel, neue oder unerfahrene MitarbeiterInnen)
Reputational risk (example: revenue authority investigation)	Reputationsrisiko (z. B.: öffentlich gewordene steuerliche Überprüfungen)
Portfolio risk (example: combination of any of the risks)	Portfolio-Risiko (z. B.: Kombination der zuvor angeführten Risiken)
*Answer options for each item*	
□ Not at all 1	□ Überhaupt nicht 1
□ 2	□ 2
□ 3	□ 3
□ 4	□ 4
□ 5	□ 5
□ 6	□ 6
□ To a great extent 7	□ In hohem Ausmaß 7
**Tax Risk (Present)**	
How would you describe your company’s tax risk profile?	Wie würden Sie das steuerliche Risikoprofil Ihres Unternehmens beschreiben?
*Answer options*	
□ Very low tax risk 1	□ Sehr niedriges steuerliches Risiko 1
□ 2	□ 2
□ 3	□ 3
□ 4	□ 4
□ 5	□ 5
□ 6	□ 6
□ Very high tax risk 7	□ Sehr hohes steuerliches Risiko 7
**Tax Risk Management System**	
Does your company have a tax risk management system (i.e. systems and/or procedures to identify and manage tax risks)?	Hat Ihr Unternehmen ein System zum steuerlichen Risikomanagament (Tax Risk Management, z. B. Systeme oder Prozesse zur Identifikation und Steuerung von steuerlichem Risiko)?
*Answer options*	
□ Yes	□ Ja
□ No	□ Nein
**Tax Risk Management Procedures**	
How important are the following systems and/or procedures to identify and manage tax risks in your company	Wie wichtig sind die folgenden Systeme/Prozesse zur Identifikation und Steuerung von steuerlichem Risiko in Ihrem Unternehmen?
*Items*	
Binding advance rulings from tax administration	Rechtsverbindlicher Auskunftsbescheid (Advance Ruling, § 118 BAO)
Advance informal agreement with tax administration	Informelle Abstimmung vorab mit der Finanzverwaltung (zB. EAS)
External advisors	Externe Beratung
Extensive documentation	Ausführliche Dokumentation
Cost analysis on possible financial penalties	Kostenalanlyse möglicher Finanzstrafen
“Smell test” based on individual experience and judgment	„Bauchgefühl“ auf Basis individueller Erfahrung und Beurteilung
Follow a benchmark firm	Einklang mit anderen Unternehmen (benchmark)
*Answer options for each item*	
□ Not important at all 1	□ Überhaupt nicht wichtig 1
□ 2	□ 2
□ 3	□ 3
□ 4	□ 4
□ 5	□ 5
□ 6	□ 6
□ Very important 7	□ Sehr wichtig 7
**Other Tax Risk Management Procedures (Open Question)**	
What other important systems exist in your company to identify and manage tax risks?	Welche weiteren wichtigen Systeme/Prozesse gibt es in Ihrem Unternehmen zur Identifikation und Steuerung von steuerlichem Risiko?
*Text fields*	
Please specify up to five other systems. 1 ___________________ 2 ___________________ 3 ___________________ 4 ___________________ 5 ___________________	Geben Sie bitte bis zu fünf weitere Systeme/Prozesse an. 1 ___________________ 2 ___________________ 3 ___________________ 4 ___________________ 5 ___________________
**Tax Risk Management Quality (Present)**	
*Items*	
Is the identification and management of tax risk in your company part of the overall risk management system?	Ist die Identifikation und Steuerung von steuerlichem Risiko in Ihrem Unternehmen Teil des allgemeinen Risikomanagement-Systems?
Is your tax risk management system well documented?	Ist Ihr steuerliches Risikomanagement-System gut dokumentiert?
Is your tax risk management system operationalized in daily business?	Ist Ihr steuerliches Risikomanagement-System im Tagesgeschäft operationalisiert?
*Answer options for each item*	
□ Not at all 1	□ Überhaupt nicht 1
□ 2	□ 2
□ 3	□ 3
□ 4	□ 4
□ 5	□ 5
□ 6	□ 6
□ To a great extent 7	□ In hohem Ausmaß 7
**Group Membership**	
Is your company part of a group?	Ist Ihr Unternehmen Teil eines Konzerns?
*Answer options*	
□ No	□ Nein
□ Yes, a group which operates only in Austria	□ Ja, ein Konzern, welcher nur in Österreich tätig ist
□ Yes, a group which operates in multiple countries	□ Ja, ein Konzern, welcher in mehreren Staaten tätig ist
**Participation in the Cooperative Compliance Program**	
Does your company participate in the horizontal monitoring project in Austria?	Nimmt Ihr Unternehmen an dem Horizontal-Monitoring-Projekt in Österreich teil?
*Answer options*	
□ Yes, because it is required by law	□ Ja, weil es vorgeschrieben war
□ Yes, because my company or group has decided to do so voluntarily	□ Ja, weil mein Unternehmen bzw. mein Konzern sich freiwillig dafür entschieden hat
□ No, because the option was not available for my company	□ Nein, da meinem Unternehmen nicht die Möglichkeit geboten wurde
□ No, because it does not meet my company’s needs	□ Nein, da es nicht den Bedürfnissen meines Unternehmens entsprach
□ No, for other reasons (please specify): _________	□ Nein, aus anderen Gründen (bitte angeben): ____________
**Time of Entering the Horizontal Monitoring Program**	
When did your company enter into the horizontal monitoring program?	Wann ist Ihr Unternehmen dem Horizontal-Monitoring-Programm beigetreten?
*Answer options*	
□ Before 2001	□ Vor 2001
□ 2001	□ 2001
□ 2002	□ 2002
[…]	[…]
□ 2016	□ 2016
□ 2017	□ 2017
**Expected Changes**	
*CC firms:* What were your company’s expectations (decrease or increase) for the following factors when entering into the horizontal monitoring program?	*CC firms:* Welche Erwartungen hatte Ihr Unternehmen bei Eintritt in das Horizontal-Monitoring-Programm für die folgenden Faktoren (Ab- oder Zunahme)?
*Control group:* Which changes (decrease or increase) of the following factors would you expect from horizontal monitoring?	*Control group:* Welche Veränderungen (Ab- oder Zunahme) der folgenden Faktoren würden Sie durch Horizontal Monitoring erwarten?
*Items*	
Tax certainty for your company	Steuersicherheit für Ihr Unternehmen
Compliance costs of your company	Rechtsbefolgungskosten für Ihr Unternehmen
Risk of penalties for your company	Risiko von Strafen für Ihr Unternehmen
Risk of penalties for individual decision makers in your company	Risiko von Strafen für einzelne entscheidende Personen in Ihrem Unternehmen
Tax risk for your company	Steuerrisiko für Ihr Unternehmen
Your own personal risk	Ihr persönliches Risiko
Reputational risk for your company	Reputationsrisiko für Ihr Unternehmen
Risk of tax litigation for your company	Risiko eines Rechtsstreits in Steuerfragen für Ihr Unternehmen
Compliance of your company with tax laws	Befolgung des Steuerrechts in Ihrem Unternehmen
Quality of the relationship between tax authorities and your company	Qualität der Beziehung zwischen Finanzverwaltung und Ihrem Unternehmen
*Answer options for each item*	
□ Strong decrease −3	□ Starke Abnahme −3
□ −2	□ −2
□ −1	□ −1
□ No change 0	□ Keine Veränderung 0
□ +1	□ +1
□ +2	□ +2
□ Strong increase +3	□ Starke Zunahme +3
**Importance of Goals**	
How important do you consider the following goals?	Wie wichtig schätzen Sie die folgenden Ziele ein?
*Items*	
Increase tax certainty for your company	Erhöhte Steuersicherheit für Ihr Unternehmen
Reduce compliance costs of your company	Geringere Rechtsbefolgungskosten für Ihr Unternehmen
Reduce the risk of penalties for your company	Geringeres Risiko von Strafen für Ihr Unternehmen
Reduce the risk of penalties for individual decision makers in your company	Geringeres Risiko von Strafen für einzelne entscheidende Personen in Ihrem Unternehmen
Reduce tax risk for your company	Geringeres Steuerrisiko für Ihr Unternehmen
Reduce your own personal risk	Geringeres persönliches Risiko für Sie
Reduce reputational risk for your company	Geringeres Reputationsrisiko für Ihr Unternehmen
Reduce the risk of tax litigation for your company	Geringeres Risiko eines Rechtsstreits in Steuerfragen für Ihr Unternehmen
Increase compliance of your company with tax laws	Verbesserte Befolgung des Steuerrechts in Ihrem Unternehmen
Improve the quality of the relationship between tax authorities and your company	Verbesserte Qualität der Beziehung zwischen Finanzverwaltung und Ihrem Unternehmen
*Answer options for each item*	
□ Not important at all 1	□ Überhaupt nicht wichtig 1
□ 2	□ 2
□ 3	□ 3
□ 4	□ 4
□ 5	□ 5
□ 6	□ 6
□ Very important 7	□ Sehr wichtig 7
**Changes—Tax Risk and Advertised Benefits**	
*CC firms:* How would you describe the actual changes (decrease or increase) of the following aspects since entering into the horizontal monitoring program?	*CC firms:* Wie würden Sie die tatsächlichen Veränderungen (Ab- oder Zunahme) der folgenden Aspekte seit dem Eintritt in das Horizontal-Monitoring-Programm beschreiben?
*Control group:* How would you describe the changes (decrease or increase) of the following aspects in your company during the last 10 [5] years? (As far as you were able to observe)	*Control group:* Wie würden Sie die Veränderungen (Ab- oder Zunahme) der folgenden Aspekte beschreiben, die während der letzten 10 [5] Jahre in Ihrem Unternehmen eingetreten sind? (Soweit Sie das beobachten konnten)
*Items*	
Tax certainty for your company	Steuersicherheit für Ihr Unternehmen
Compliance costs of your company	Rechtsbefolgungskosten für Ihr Unternehmen
Risk of penalties for your company	Risiko von Strafen für Ihr Unternehmen
Risk of penalties for individual decision makers in your company	Risiko von Strafen für einzelne entscheidende Personen in Ihrem Unternehmen
Tax risk for your company	Steuerrisiko für Ihr Unternehmen
Your own personal risk	Ihr persönliches Risiko
Reputational risk for your company	Reputationsrisiko für Ihr Unternehmen
Risk of tax litigation for your company	Risiko eines Rechtsstreits in Steuerfragen für Ihr Unternehmen
Compliance of your company with tax laws	Befolgung des Steuerrechts in Ihrem Unternehmen
Quality of the relationship between tax authorities and your company	Qualität der Beziehung zwischen Finanzverwaltung und Ihrem Unternehmen
*Answer options for each item*	
□ Strong decrease −3	□ Starke Abnahme −3
□ −2	□ −2
□ −1	□ −1
□ No change 0	□ Keine Veränderung 0
□ +1	□ +1
□ +2	□ +2
□ Strong increase +3	□ Starke Zunahme +3
**Changes—Tax Risk Sources**	
*CC firms:* How have the following components of tax risk changed (decrease of increase) since entering into the horizontal monitoring program?	*CC firms:* Wie haben sich die folgenden Kompenenten des steurlichen Risikos seit dem Eintritt in das Horizontal-Monitoring-Programm verändert (Ab- oder Zunahme)?
*Control group:* How have the following components of tax risk changed (decrease or increase) in your company during the last 10 [5] years? (As far as you were able to observe)	*Control group:* Wie haben sich die folgenden Komponenten des steuerlichen Risikos in Ihrem Unternehmen während der letzten 10 [5] Jahre verändert (Ab- oder Zunahme)? (Soweit Sie das beobachten konnten)
*Items*	
Transactional risk (examples: acquisitions, mergers)	Transaktionsrisiko (z. B.: Akquisitionen, Verschmelzungen)
Operational risk (examples: new business ventures, new operating models, new operating structure)	Operatives Risiko (z. B.: neue Geschäftsfelder, neue Prozesse, neue Strukturen)
Compliance risk (examples: weak records and controls, legislative changes)	Compliance-Risiko (z. B.: Schwächen bei Aufzeichnungen oder Kontrollen, gesetzliche Änderungen)
Financial accounting risk (examples: changes in systems and policies)	Rechnungslegungsrisiko (z. B.: Änderungen in Rechnungslegungsprozessen oder -politik)
Management risk (examples: changes in personnel, new/inexperienced resources)	Management-Risiko (z. B.: Personalwechsel, neue oder unerfahrene MitarbeiterInnen)
Reputational risk (example: revenue authority investigation)	Reputationsrisiko (z. B.: öffentlich gewordene steuerliche Überprüfungen)
Portfolio risk (example: combination of any of the risks)	Portfolio-Risiko (z. B.: Kombination der zuvor angeführten Risiken)
*Answer options for each item*	
□ Strong decrease −3	□ Starke Abnahme −3
□ −2	□ −2
□ −1	□ −1
□ No change 0	□ Keine Veränderung 0
□ +1	□ +1
□ +2	□ +2
□ Strong increase +3	□ Starke Zunahme +3
**Changes—Tax Risk Management Quality**	
*CC firms:* How have the following aspects changed (decrease or increase) since entering into the horizontal monitoring program?	*CC firms:* Wie haben sich die folgenden Aspekte seit dem Eintritt in das Horizontal-Monitoring-Programm verändert (Zu- oder Abnahme)?
*Control group:* How have the following aspects changed (decrease or increase) in your company during the last 10 [5] years? (As far as you were able to observe)	*Control group:* Wie haben sich die nachstehenden Aspekte in Ihrem Unternehmen während der letzten 10 [5] Jahre verändert (Ab- oder Zunahme)? (Soweit Sie das beobachten konnten)
*Items*	
Degree to which the general risk management system is formalised (i.e. well documented)	Ausmaß der Formalisierung des generellen Risikomanagement-Systems (zB. ausdrücklichen Dokumentation)
Quality of the general risk managament system	Qualität des generellen Risikomanagement-Systems
Degree to which the tax risk management system is formalised (i.e. well documented)	Ausmaß der Formalisierung des steuerlichen Risikomanagement-Systems (zB. ausdrücklichen Dokumentation)
Quality of the tax risk management system	Qualität des steuerlichen Risikomanagement-Systems
Degree to which tax risk is included in the general risk management system	Ausmaß, in dem das steuerliche Risiko im generellen Risikomanagement-System erfasst ist
*Answer options for each item*	
□ Strong decrease −3	□ Starke Abnahme −3
□ −2	□ −2
□ −1	□ −1
□ No change 0	□ Keine Veränderung 0
□ +1	□ +1
□ +2	□ +2
□ Strong increase +3	□ Starke Zunahme +3
**Other Changes (Open Question)**	
*CC firms:* What other important tax-related changes has your company experienced since entering the horizontal monitoring program?	*CC firms:* Welche weiteren wichtigen steuerbezogenen Veränderungen konnte Ihr Unternehmen seit dem Eintritt in das Horizontal-Monitoring-Programm feststellen?
*Control group:* What other important tax-related changes has your company experienced during the last 5 years? (As far as you were able to observe)	*Control group:* Welche weiteren wichtigen steuerbezogenen Veränderungen konnte Ihr Unternehmen während der letzten 10 [5] Jahre feststellen? (Soweit Sie das beobachten konnten)
*Text fields*	
Please specify up to five other positive/negative changes. Positive changes: 1 ___________________ 2 ___________________ 3 ___________________ 4 ___________________ 5 ___________________ Negative changes: 1 ___________________ 2 ___________________ 3 ___________________ 4 ___________________ 5 ___________________	Geben Sie bitte bis zu fünf weitere positive bzw. negative Veränderungen an. Positive Veränderungen: 1 ___________________ 2 ___________________ 3 ___________________ 4 ___________________ 5 ___________________ Negative Veränderungen: 1 ___________________ 2 ___________________ 3 ___________________ 4 ___________________ 5 ___________________
**Group Parent Residence**	
In which country is the ultimate group parent resident?	In welchem Land ist die Konzernobergesellschaft ansässig?
*Answer options*	
□ Australia	□ Australien
□ Austria	□ Österreich
□ United Kingdom	□ Vereinigtes Königreich
□ Germany	□ Deutschland
□ France	□ Frankreich
□ United States	□ Vereinigte Staaten von Amerika
□ Afghanistan	□ Afghanistan
[…]	[…]
□ Zimbabwe	□ Zimbabwe
**Local Sales**	
Approximately, what are the annual total sales (turnover) of your company in Austria?	Wie hoch ist ca. der jährliche Umsatz Ihres Unternehmens in Österreich?
*Answer options*	
□ Less than 0.7 million euro	□ Weniger als 0,7 Millionen Euro
□ 0.7–10 million euro	□ 0,7–10 Millionen Euro
□ 10–40 million euro	□ 10–40 Millionen Euro
□ 40–250 million euro	□ 40–250 Millionen Euro
□ 250–1000 million euro	□ 250–1000 Millionen Euro
□ More than 1 billion euro	□ Mehr als 1 Milliarde Euro
□ No answer	□ Keine Angabe
**Worldwide Sales**	
Approximately, what are the annual total sales (turnover) of your group worldwide?	Wie hoch ist ca. der weltweite jährliche Konzernumsatz?
*Answer options*	
□ Less than 0.7 million euro	□ Weniger als 0,7 Millionen Euro
□ 0.7–10 million euro	□ 0,7–10 Millionen Euro
□ 10–40 million euro	□ 10–40 Millionen Euro
□ 40–250 million euro	□ 40–250 Millionen Euro
□ 250–1000 million euro	□ 250–1000 Millionen Euro
□ More than 1 billion euro	□ Mehr als 1 Milliarde Euro
□ No answer	□ Keine Angabe
**Jurisdictions**	
Approximateley, how many tax jurisdictions is your company subjected to annually?	Mit dem Steuerrecht wie vieler unterschiedlicher Länder setzt sich Ihr Unternehmen ca. pro Jahr auseinander?
*Answer options*	
□ One tax jurisdiction (domestic only)	□ Steuerrecht eines Landes (nur Inland)
□ 2–5 tax jurisdictions	□ Steuerrecht von 2–5 Ländern
□ 6–10 tax jurisdictions	□ Steuerrecht von 6–10 Ländern
□ 11–20 tax jurisdictions	□ Steuerrecht von 11–20 Ländern
□ More than 21 tax jurisdictions	□ Steuerrecht von mehr als 21 Ländern
□ No answer	□ Keine Angabe
**Stock Exchange**	
Is your company or any other company in the group publicly listed on a stock exchange?	Ist Ihr Unternehmen oder ein anderes Konzernunternehmen an der Börse notiert?
*Answer options*	
□ Yes	□ Ja
□ No	□ Nein
□ No answer	□ Keine Angabe
**Tax Compliance (Firm)**	
In my company, tax compliance means …	In meinem Unternehmen bedeutet steuerliche Compliance …
*Items*	
… following the letter of tax laws	… dem Wortlaut des Steuerrechts zu folgen
… following the spirit of tax laws	… dem Sinn des Steuerrechts zu folgen
*Answer options for each item*	
□ Strongly disagree 1	□ Stimme überhaupt nicht zu 1
□ 2	□ 2
□ 3	□ 3
□ 4	□ 4
□ 5	□ 5
□ 6	□ 6
□ Strongly agree 7	□ Stimme stark zu 7
**Position**	
What is your position in your company?	Welche Position haben Sie in Ihrem Unternehmen inne?
*Answer options*	
□ Chief Executive Officer	□ Vorstandsvorsitzende/r, Geschäftsführer/in (CEO)
□ Chief Financial Officer	□ Finanzvorstand (CFO)
□ Head of Accounting	□ Leiter/in Rechnungswesen
□ Tax Director	□ Leiter/in Steuerabteilung
□ Tax Manager	□ Steuerexperte/in
□ Assistant Tax Manager	□ Steuersachbearbeiter/in
□ No answer	□ Keine Angabe
□ Other (please specify): __________	□ Andere (bitte angeben):__________
**Tax Compliance (Personal)**	
To me personally, tax compliance means …	Für mich persönlich bedeutet steuerliche Compliance …
*Items*	
… following the letter of tax laws	… dem Wortlaut des Steuerrechts zu folgen
… following the spirit of tax laws	… dem Sinn des Steuerrechts zu folgen
*Answer options for each item*	
□ Strongly disagree 1	□ Stimme überhaupt nicht zu 1
□ 2	□ 2
□ 3	□ 3
□ 4	□ 4
□ 5	□ 5
□ 6	□ 6
□ Strongly agree 7	□ Stimme stark zu 7
**Personal Risk Attitude**	
How would you describe your personal risk attitude?	Wie würden Sie Ihre persönliche Risikoneigung einschätzen?
*Answer options*	
□ Very risk-averse 1	□ Sehr risikoscheu 1
□ 2	□ 2
□ 3	□ 3
□ 4	□ 4
□ 5	□ 5
□ 6	□ 6
□ Very risk-seeking 7	□ Sehr risikofreudig 7
**Age**	
What is your age?	Bitte geben Sie Ihr Alter an
*Answer options*	
□ Under 25	□ Unter 25
□ 25–34	□ 25–34
□ 35–44	□ 35–44
□ 45–54	□ 45–54
□ 55–64	□ 55–64
□ Over 64	□ Über 64
□ No answer	□ Keine Angabe
**Gender**	
What is your gender?	Bitte geben Sie Ihr Geschlecht an
*Answer options*	
□ Female	□ Weiblich
□ Male	□ Männlich
□ No answer	□ Keine Angabe

## Footnotes

1 Cooperative compliance (“Horizontal Monitoring”, referring to equal footing) was first introduced in the Netherlands in 2005, where a pilot project started with 20 large businesses (Stevens et al. 2012). Subsequently, the program expanded and soon included SMEs. Both businesses and authorities are in a trusting relationship with shared responsibilities in order to efficiently apply tax laws (Stevens et al. 2012; de Widt 2017). Currently, approximately 3800 businesses participate (de Widt and Oats 2017).

2 Even though also partnerships could participate, only corporations (including GmbH Co KG) did.

3 To the best of our knowledge, these 13 groups have remained in HM also after the pilot phase.

4 Long term effective tax rate per firm is calculated as the sum of income taxes per firm related to the sum of earnings before taxes per firm, over the years 2011 to 2018, from consolidated profit and loss statements, as available from SABINA database. For one firm, no tax data were available.

5 Sections 153a–153g Bundesabgabenordnung.

6 Original source no longer accessible.

7 One firm opted out of the HM regime in 2017.

8 Such selection seems preferable to a broader dissemination because firms in the Tax Policy Group can be expected to be particularly comparable to HM firms in terms of size and engagement in tax matters.

9 Our survey design cannot ensure that the addressees of the survey actually participated themselves. Delegation to employees is possible but not expected.

10 Interviews were conducted in 2015 with Hubert Woischitzschläger, Karl Hofbauer and Tina Ehrke-Rabl.

11 We did not achieve a balanced distribution of years in the control group. Nine participants of the control group answered change-related questions for the past 5 years, 22 for the past 10 years.
